# A synthetic consortium of 100 gut commensals modulates the composition and function in a colon model of the microbiome of elderly subjects

**DOI:** 10.1080/19490976.2021.1919464

**Published:** 2021-05-16

**Authors:** Marta Perez, Alexandra Ntemiri, Huizi Tan, Hugh M. B. Harris, Henrik M. Roager, Céline Ribière, Paul W. O’Toole

**Affiliations:** a School of Microbiology and APC Microbiome Ireland, University College Cork, Cork, Ireland; b Department of Nutrition, Exercise and Sports, University of Copenhagen, Frederiksberg, Denmark

**Keywords:** Gut microbiome, synthetic microbiome, consortium, antibiotics, alpha-diversity, elderly, branched chain amino acids, BCAA

## Abstract

Administration of cultured gut isolates holds promise for modulating the altered composition and function of the microbiota in older subjects, and for promoting their health. From among 692 initial isolates, we selected 100 gut commensal strains (MCC100) based on emulating the gut microbiota of healthy subjects, and retaining strain diversity within selected species. MCC100 susceptibility to seven antibiotics was determined, and their genomes were screened for virulence factor, antimicrobial resistance and bacteriocin genes. Supplementation of healthy and frail elderly microbiota types with the MCC100 in an *in vitro* colon model increased alpha-diversity, raised relative abundance of taxa including *Blautia luti, Bacteroides fragilis*, and *Sutterella wadsworthensis*; and introduced taxa such as *Bifidobacterium* spp. Microbiota changes correlated with higher levels of branched chain amino acids, which are health-associated in elderly. The study establishes that the MCC100 consortium can modulate older subjects’ microbiota composition and associated metabolome *in vitro*, paving the way for pre-clinical and human trials.

## Introduction

The elderly proportion of the global population (65 years or older) is increasing faster than previously, and is expected to rise from 10% to 17% by 2050.^1^ The health care of older adults is now a global public challenge that includes the promotion of health span and independent living through the reduction of frailty and comorbidities. Hence, there is a social commitment to formulate evidence-based strategies that support healthy aging. The gut microbiota is a modifiable environmental factor that has the potential to affect the health of older individuals.^2^

The gut microbiota structure changes gradually during aging and varies considerably between older individuals. Reductions in alpha diversity, lowered abundance of subdominant taxa, and the depletion of bifidobacteria and fiber-responsive taxa occur in the gut microbiota of older subjects compared to younger adults.^3,4^ Some of these changes are accelerated by consuming a restricted-range diet,^5^ but fiber-responsive taxa are difficult to restore by fiber supplementation.^6^ Age-related alteration of the gut microbiota is associated with constitutive low-grade inflammation, and loss of the gut barrier function which contributes to frailty and increased susceptibility to pathobiont infections.^7^

The lowest diversity microbiota is found in older subjects that live in long-term residential care and who consume a restricted diet that is less diverse, low in fiber and enriched in saturated fat. The corresponding microbiota profile co-varies with increased frailty and co-morbidity measurements and correlates with higher pro-inflammatory marker levels.^3,5^ Other studies confirm that alterations in the age-related gut microbiota co-vary with physical frailty and the reduction of alpha-diversity correlates with biological age.^8–11^ Therefore, there is considerable interest in developing microbiota restoration as a therapeutic strategy in older people. Potential strategies include the use of prebiotics, probiotics and synbiotics, but they have limited ability to produce a dramatic change in the composition of the microbiota.^4^ We have shown that the administration of a mix of five prebiotics to frail older subjects for 6 months resulted in some alteration of the relative abundance of selected taxa, but only modest improvements in inflammatory markers.^6^ Fecal microbiota transplantation immediately alters the gut microbiota, for example, in the treatment of recurrent *Clostridioides difficile* infections (CDI).^12^ However, fecal bacteriotherapy for older subjects is made more difficult by the challenge of donor screening issues,^13^ and elderly recipients are by definition at-risk individuals. Novel forms of biotherapies with synthetic microbiotas – where the exact microbiota configuration is defined – are now in development. These microbial mixtures would ideally act as a community or network, and simulate the diversity and robustness of a healthy microbial ecosystem. They can be reproduced and customized and the antimicrobial susceptibilities of microbes can be identified so that antibiotics can be reliably used if needed. Moreover, this technique could be conceived as a preventive treatment.^14^ Artificial microbial consortia have been shown to be efficient against CDI in humans,^15,16^ and are under investigation for application in other conditions.^17–19^

Advances in culturomics have allowed the isolation of previously “uncultivable” gut microorganisms,^20–22^ assembling catalogs of strains that could be used to restore intestinal dysbiosis. The description of the healthy core microbiota, plus detailed definition of age-associated changes in healthy and frail older people, allows rational design of synthetic microbial mixtures that can modulate microbiota alteration in elderly subjects. Here we sought to develop an artificial microbial consortium that could be used to rectify the low diversity gut microbiota of elderly subjects. From a large collection of gut commensal strains that we isolated, we selected 100 strains that are prevalent and abundant in the microbiota of healthy subjects. The ability of this consortium to modulate microbiota composition and diversity, and the metabolome, was investigated in an *in vitro* model.

## Results

### Development of a defined microbiota consortium, the MCC100

A large catalog termed the Microbiome Culture Collection (MCC) was established by anaerobic isolation of 692 commensal microbes from fecal samples of healthy donors (Supplementary file 1). Genera and species isolated are listed in Supplementary Table S1a. To choose isolates for emulating the configuration of a healthy gut microbiota, the main selection criteria used were prevalence and abundance in the core human gut microbiota composition, and phylogenetic diversity of the species; 15 major studies were reviewed (Supplementary Table S1b). The reported abundance of the microbial groups was integrated into the MCC100 design. First, we selected isolates whose species or genus has been described in at least one of the 15 reviewed studies (57 species). We further added species not explicitly reported but classified (according to our previous fine-detail microbiome analysis)^5^ in the Core, Core-Reduced Core or Diversity Associated iBBiG-OTU groups (6 species from the genera *Bacteroides, Blautia* and *Clostridium* XIVa and XIVb); species described as common gut members by other studies (3 species from the genus *Enterococcus* and the species *Propionibacterium acnes* and *Ruminococcus bicirculans*); species with potential probiotic properties (four species from the former genus *Lactobacillus*); and two species that may increase the diversity of *Clostridium* cluster XIVa (*Clostridium saccharolyticum* and *Clostridium celerecrescens*) (Supplementary Table S1a). In total, 74 commensal species belonging to 19 families and 35 genera were selected.

Twenty-two of the 74 species were singleton strains. For the other 51 species, RAPD-PCR analysis was performed upon a subset of 171 isolates: up to 4 isolates per species from different donors were analyzed when possible. 117 different RAPD-PCR profiles were obtained (Supplementary Table S2 and Supplementary Figure S1). In total, a collection of 139 different strains was thus identified. Finally, 100 different strains were selected, reflecting species with high reported abundance in the gut microbiota in the surveyed literature. In order to maximize the genetic diversity of the consortium, multiple strains from the same species were selected aiming for lowest genetic similarity according to dendrograms constructed from the RAPD-PCR patterns (Supplementary Figure S2).

**Figure 1.**
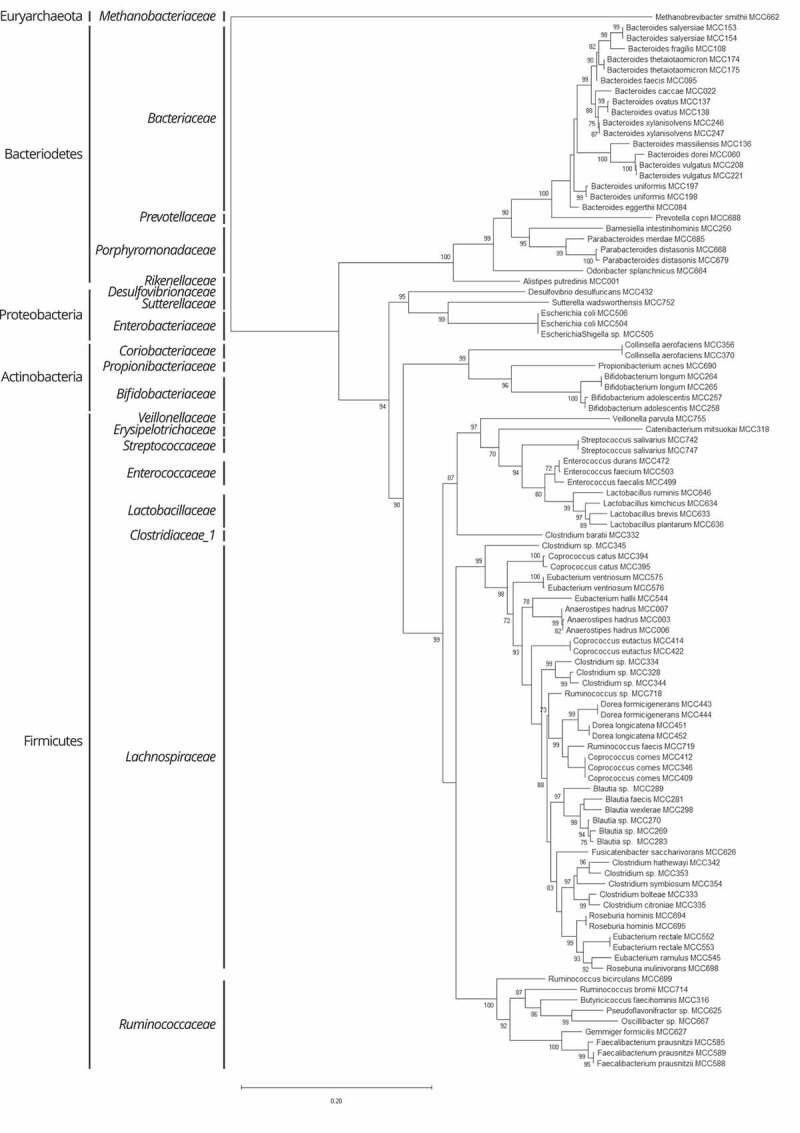
Phylogeny of MCC100 strains. Phylogenetic maximum likelihood tree using the Generalized Time-Reversible model with CAT approximation with 20 rate categories inferred from the 16S rRNA gene. The tree is rooted on the domain Archaea for illustrative purposes. Local support values superior or equal to 70% are displayed. Taxonomic classification at family and phylum levels is indicated on the left side of the tree

The resulting artificial consortium was termed the Microbiome Culture Collection 100 (MCC100). Taxonomic analysis (Supplementary Table S3 and Supplementary Figure S3) of the consortium graphically illustrates the phylogenetic breadth of the MCC100 comprizing 75 different species, 13 being classified up to genus level representing new or poorly characterized species (Figure 1). The genomes of the MCC100 strains were sequenced. Thirty-seven are classified among the most recent list of the HMP “Most wanted” taxa for genome sequencing priority (Supplementary Table S4).

### MCC100 strain characterization

**Figure 2.**
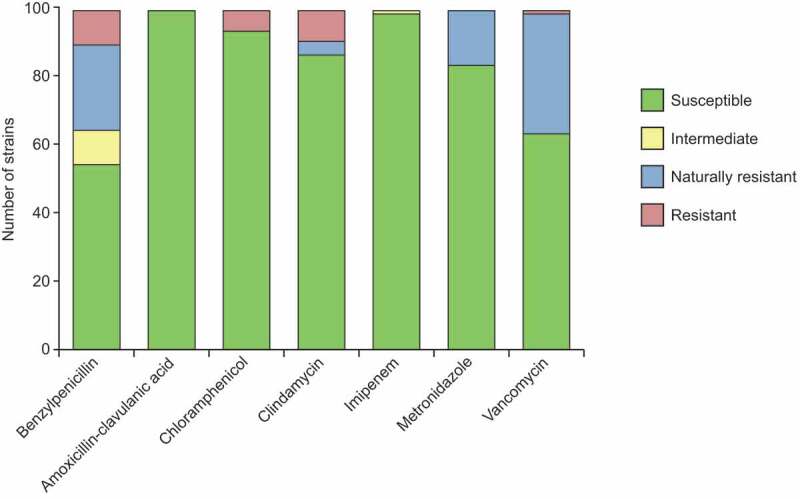
Antimicrobial susceptibility of the MCC100 bacterial strains. The number of susceptible, intermediate resistant, resistant and naturally resistant strains is indicated for each antibiotic tested. MIC values were determined by gradient strip method and the results interpreted according to EUCAST guidelines. Vancomycin was only tested in the Gram-positive strains as indicated by EUCAST and intrinsic resistance was assumed for the non-tested strains for the graphical representation

To evaluate the safety of the selected strains for eventual administration to humans, we determined their MIC values for a panel of seven antibiotics and interpreted the resistance based on EUCAST cutoff values. The *Methanobrevibacter smithii* MCC662 isolate could not be tested due to its inability to grow on plates. The bacterial susceptibility data are presented in Figure 2 and Supplementary Table S5a. Benzylpenicillin had the lowest inhibition activity with 35 resistant strains. However, the combination of a penicillin and a beta-lactamase inhibitor was very effective since amoxicillin-clavulanate inhibited the growth of all 99 bacterial strains. Imipenem inhibited 98 strains, with *Faecalibacterium prausnitzii* MCC585 showing intermediate resistance, but this strain was only tested on YCFA plates due to the lack of growth in the EUCAST-recommended testing medium. Moreover, 93 strains were sensitive to chloramphenicol, 86 to clindamycin, 83 to metronidazole and 63 out of 68 to vancomycin. Nevertheless, resistance to some antibiotics has been reported to be intrinsic in specific bacterial groups (Supplementary Table S5a, MIC values colored in blue). Indeed, while 48 strains had no resistance to any antimicrobial tested, 31 strains had endogenous resistance to up to 4 antibiotics. All 99 bacterial strains could be inhibited by using amoxicillin-clavulanate or combinations of imipenem with any of the other antibiotics tested, or metronidazole with chloramphenicol (Supplementary Table S5b). The aerobic strains were susceptible to the additional antimicrobials tested by disk diffusion method (Supplementary Table S5c).

We sequenced the MCC100 genomes and screened them for the presence of genes involved in antibiotic resistance, virulence and bacteriocin production. We annotated 278 sequences belonging to 88 different antibiotic resistance genes across 64 of the MCC100 strains, which are predicted to encode resistance to 13 drug classes (Supplementary Table S6a). The number of genes ranged from 1 to 49 per strain. While most of the genomes harbored one or two predicted antibiotic resistance genes, the three *Escherichia/Shigella* genomes harbored more than half of the annotated sequences (141) encoding the majority of multidrug transport genes identified here, and all the fosfomycin, peptide antibiotics, and sulfonamide resistance proteins. Tetracycline resistance genes were present in 40 strains of *Bacteroidetes* and *Firmicutes*, being the most widespread resistance function. Mupirocin and rifampycin resistance genes were exclusively found in *Actinobacteria*. Gene prediction and phenotypic resistance were correlated for 18 out of 35 benzylpenicillin and 6 out of 13 clindamycin resistant strains (Supplementary Table S6a). No genetic basis was identified for the vancomycin and chloramphenicol resistant strains. In contrast, three strains contained vancomycin resistance genes in their genomes even though they were susceptible; and the genomes of three enterococcal and three enterobacterial strains susceptible to gentamicin and streptomycin harbored aminoglycosides resistance genes.

One hundred and seventy-four different putative virulence factors were annotated in 474 genes across 61 MCC100 strains (Supplementary Table S6b). Gene counts per isolate ranged from 1 to 114. The *Escherichia/Shigella* genomes contained 317 genes. The most common genes encoded putative capsular polysaccharide biosynthesis proteins (26 strains), followed by protease genes (19 strains) and catalase encoding genes (13 strains), which are found in other commensal microbes. Thirty putative bacteriocin loci were annotated in 12 MCC100 genomes. Most of these predicted bacteriocins were found in the genomes of the facultatively aerobic strains and belonged to class II (Supplementary Table S6c).

### Effect of supplementing elderly microbiota types with the MCC100 in an in vitro colon model

The capacity of the MCC100 to influence the microbiota of elderly was tested in an *in vitro* colon model. The system was inoculated with fecal samples from six elderly donors – three healthy individuals living in the community (CM) and three frail subjects in long-stay care (LS). Each sample was run with and without the MCC100 supplementation, and microbiota composition and diversity were studied by 16S rRNA gene sequencing analysis.

First, we confirmed that the donor inocula reflected the differences between CM and LS microbiota types described in our previous studies.^5^ CM samples clustered closer together and separated from the LS samples based on the PCoA of the weighted and unweighted UniFrac (Supplementary Figure S4). CM subjects harbored microbiota with higher alpha-diversity indexes than LS donors (Supplementary Figure S5a). Additionally, we searched for the presence of MCC100 taxa in the fecal samples by comparing the full-length 16S rRNA genes of the 100 strains against the V3/V4 16S rRNA gene reads. An average of 75 microbes close to MCC100 taxa were present in CM samples whereas 61 were detected in LS (Supplementary Figure S5b), suggesting MCC100 supplementation would add some of the missing taxa in the LS compared to CM subjects, as well as other taxa absent in both microbiota types. Furthermore, measurement of the alpha-diversity indices of the MCC100 inoculum revealed similar Shannon (4.6) and Simpson (0.92) values to those obtained for the fecal samples, indicating MCC100 would simulate the diversity level of a human microbiota (Supplementary Figure S6a).

**Figure 3.**
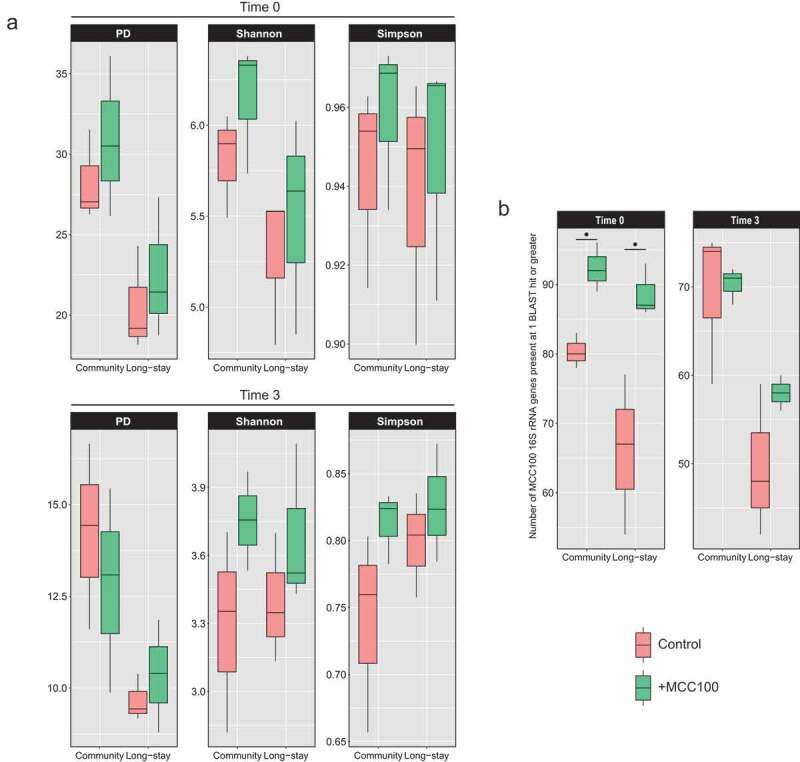
(a) Alpha-diversity indexes of control (red) and MCC100 supplemented (green) fermentations for community and long-stay microbiota types at time 0 and time 3. (b) Presence of MCC100 species across fermentation samples. BLAST results of the MCC100 16S rRNA gene full-length sequences against the V3/V4 16S rRNA gene reads of the fecal samples were filtered at 98.7% identity and 90% coverage. MCC100 taxa with one hit or greater were considered as present in the sample. Statistically significant differences were determined using Mann-Whitney test (one-tailed) (∙ exact *p*-value = 0.05)

The microbiota alpha-diversity of the fermentation samples was analyzed. At time 0 the supplementation of the CM and LS fecal samples with the MCC100 resulted in the expected numerical rise of the alpha-diversity indices (Figure 3a). After 3 days running and as a consequence of the microbial adaptation to the fermenter conditions, the alpha-diversity typically drops compared with time 0 values. However, CM and LS inocula supplemented with the MCC100 showed higher Shannon and Simpson indexes values than control groups (CM control 3.2 and 0.74 *vs* CM+MCC100 3.6 and 0.81; LS control 3.4 and 0.79 *vs* LS+MCC100 3.7 and 0.82 for Shannon and Simpson indexes respectively). Despite the indices showing the same trend, the differences were not statistically significant, likely because of low power due to small sample size. However, comparisons of control and MCC100 supplemented fermenter data after aggregating CM and LS samples returned significant difference for the Shannon index at time 3 (*p*-value = 0.032) (Supplementary Figure S6b). This supports the hypothesis that administration of the MCC100 adds unique taxa or modifies the abundances of the populations in complex communities (such is in the human gut).

Analysis of the presence of MCC100 taxa in fermentation samples showed an increase in the number of MCC100 taxa in the CM and LS supplemented samples at time 0 (CM control 80 *vs* CM+MCC100 92, *p*-value = 0.05; LS control 66 *vs* LS+MCC100 88, *p*-value = 0.05), confirming MCC100 supplementation adds taxa absent in the CM and LS microbiota types. After the taxon loss during the fermentation run, a numerical difference in MCC100-derived 16S reads was observed between MCC100 supplemented and the control for the LS samples only (LS control 49 *vs* LS+MCC100 58) (Figure 3b).

**Figure 4.**
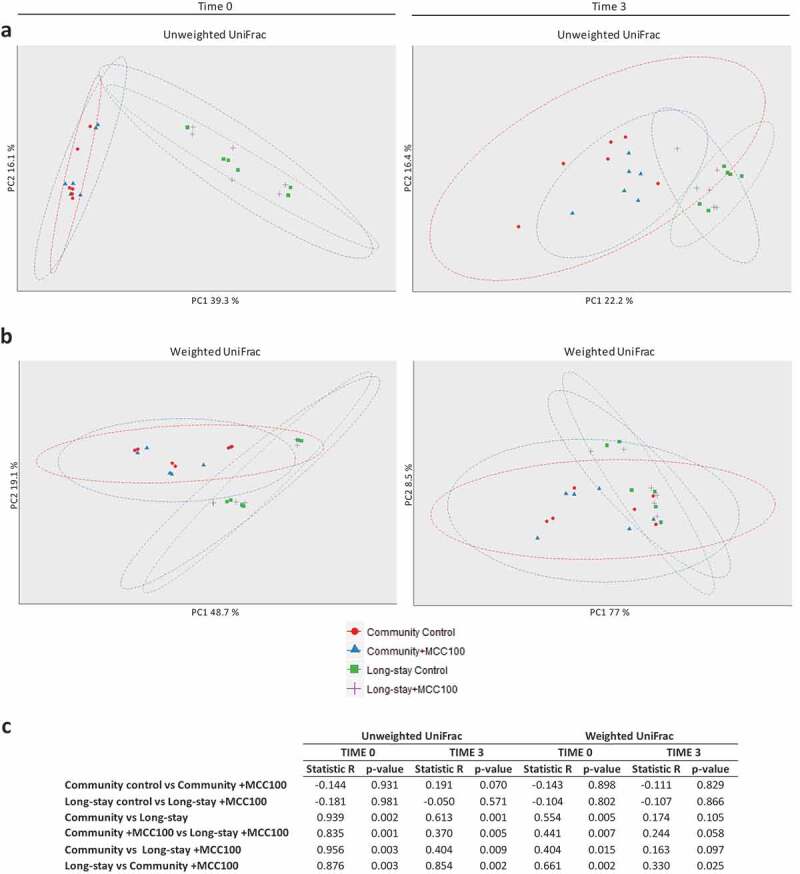
(a, b) Principal coordinate analysis (PCoA) differentiates microbiota patterns for MCC100 supplemented (blue and purple) and control (red and green) fermentations for community (red and blue) and long-stay (green and purple) microbiota types on (a) unweighted and (b) weighted UniFrac distance matrices. (c) Analysis of similarities (ANOSIM) based on unweighted and weighted UniFrac distance matrices

Differences in microbiota composition and relative abundance between CM and LS fermentations supplemented or not with the MCC100 were analyzed by PCoA on unweighted and weighted UNIFRAC distance matrices (Figure 4). Detailed results are described in Supplementary file 1. At time 0, CM and LS samples differed both in taxon composition and relative abundance. Microbiota type was the main driver of variability between the samples while MCC100 supplementation did not significantly separate CM and LS with respect to their controls. The same separation profiles were observed at time 3. However, microbiota type explained a lower proportion of variability, indicating a selective effect of fermenter conditions. R values derived from ANOSIM test suggested CM and LS samples supplemented with the MCC100 were more similar in composition than their non-supplemented control groups.

Analysis of the microbiota profiles in CM and LS samples with and without the MCC100 addition showed the microbiota was initially dominated (relative abundance ≥1%) by 16 families in CM samples and 14 in LS with and without the addition of MCC100 (Supplementary Figure S7). After the culture period, the number of dominant families reduced to six in CM control samples, nine in CM+MCC100, five in LS control and seven in LS+MCC100, indicating a selective effect due to fermentation conditions but that MCC addition reduces taxon loss in both microbiota types. Eight and seven dominant families were detected in the fermentation of the MCC100 alone at time 0 and time 3, respectively.

**Figure 5.**
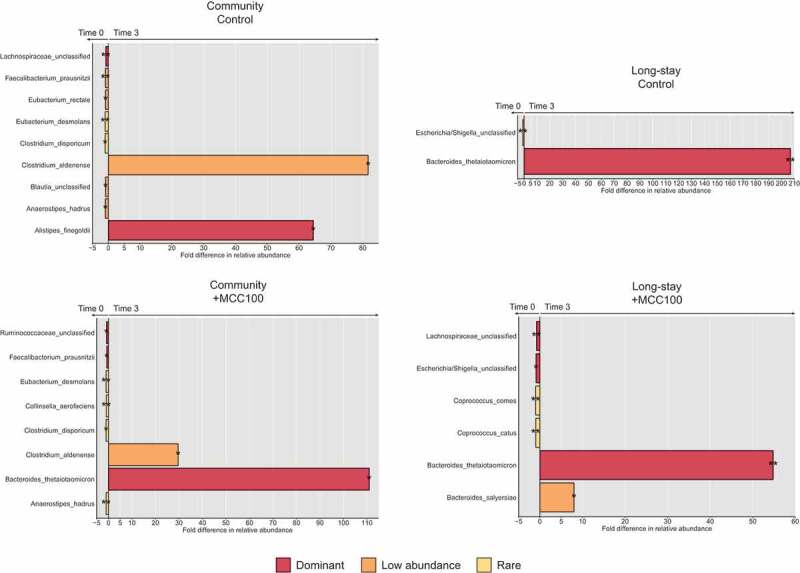
Fold differences in relative abundance of bacterial species in community (a) and long-stay (b) microbiota types supplemented with the MCC100 or control showing significant differences between time 0 and time 3. Bacterial species were classified as dominant (relative abundance ≥ 1%), low abundant (relative abundance between 0.1 and 1%) or rare (relative abundance ≤ 0.1%). Statistically significant differences were determined using Kruskal–Wallis test (*p*-value<0.05), followed by Dunn’s post-hoc test (*p-value <0.05, ** p-value *<*0.005)

Next, we examined the differences in relative abundance of the microbiota species between time 0 and time 3 in CM and LS samples with the MCC100 and control (Figure 5). Changes in the relative abundance of taxa due to fermentation were observed in both the MCC100 supplemented and control groups (Supplementary file 1). These included reductions of *F. prausnitzii* abundance in CM samples or unclassified *Escherichia/Shigella* in LS samples. However, *F. prausnitzii* remained among the dominant taxa in the MCC100 supplemented condition while it was at low abundance in the control (0.9% control *vs* 3.1% +MCC100 at time 3), suggesting MCC100 sustains the relative abundance of *F. prausnitzii* in the CM microbiota type. Similarly, while unclassified *Escherichia/Shigella* had a low abundance in the control condition, it remained among the dominant taxa with the MCC100 addition (0.5% control *vs* 1.2% +MCC100 at time 3).

Notably, comparisons of relative abundances between control and supplemented condition when aggregating CM and LS samples revealed that MCC100 affected the proportion of the following species: at time 0, *Bacteroides fragilis* (0.09% control *vs*0.32% +MCC100, *p*-value = 0.041), *Bacteroides salyersiae* (0.01% *vs* 0.06%, *p*-value = 0.044) and *B. thetaiotaomicron* (0.10% *vs* 0.35%, *p*-value = 0.002) populations increased with the MCC100 addition. At time 3, the relative abundances of *B. fragilis* (0.05% *vs* 1.30%, *p*-value = 0.004), *Blautia luti* (0.04% *vs* 0.10%, *p*-value = 0.041) and *S. wadsworthensis* (0.17% *vs* 1.20%, *p*-value = 0.030) were higher when elderly microbiota were cultured with the MCC100.

**Figure 6.**
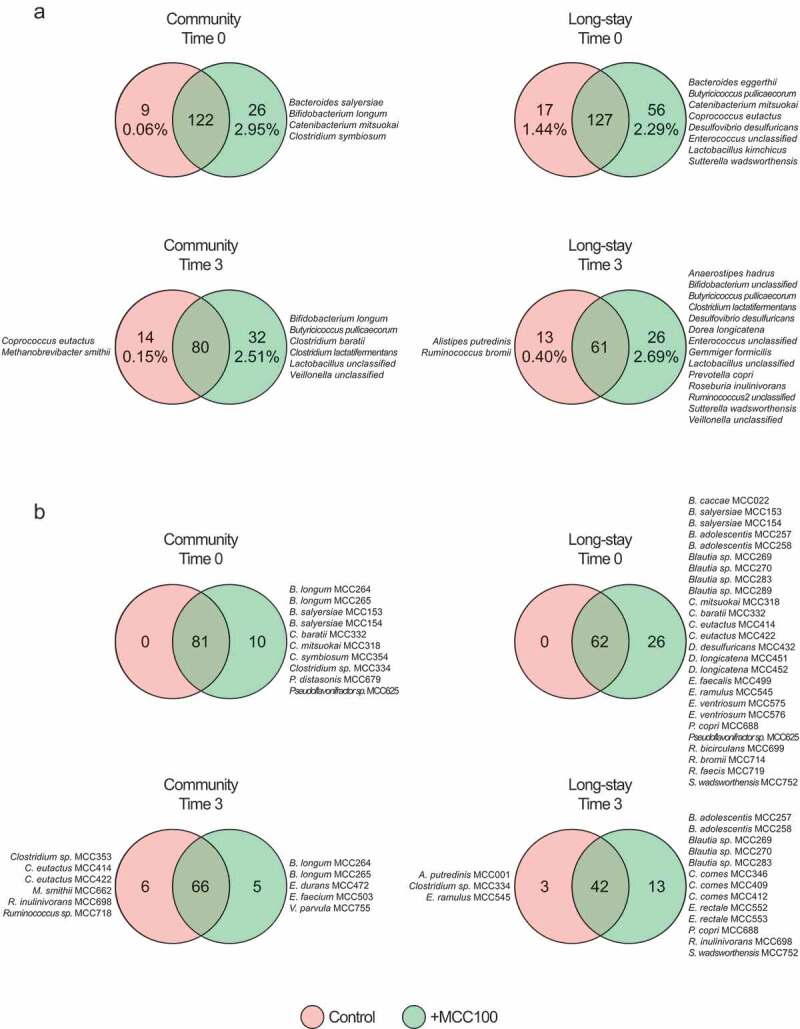
(a) Shared and unique bacterial species identified by 16S rRNA gene sequencing analysis at time 0 and time 3 in MCC100 supplemented (green) and control (red) fermentations for community and long-stay microbiota types (species that were present in both technical replicates and in at least 50% of the samples in each group). (b) MCC100 taxa identified as indicated in Figure 3 in the same samples

Unique and shared species between control and MCC100 supplemented CM and LS samples were studied (Supplementary Table S7). Consistent with higher alpha-diversity indices, a greater number of species was detected in the MCC100 supplemented CM and LS samples compared with control groups at time 0 and time 3 (Figure 6a). The co-culture of CM microbiota and MCC100 returned 32 unique species with 2.5% aggregated relative abundance. These unique taxa included *Bifidobacterium longum, Butyricicoccus pullicaecorum, Clostridium baratii, Clostridium lactatifermentas, Lactobacillus* unclassified, and *Veillonella* unclassified which could be members of the added MCC100. *Coprococcus eutactus* and *M. smithii* were only detected in the CM control samples at time 3 despite the same species being present in the MCC100. The LS samples inoculated with the MCC100 harbored 26 unique species at time 3 (2.7% aggregated relative abundance). These taxa comprised *B. pullicaecorum, C. lactatifermentans, Lactobacillus* unclassified, *Veillonella* unclassified (the last four were commonly detected in CM supplemented samples at time 3), *Desulfovibrio desulfuricans, Enterococcus* unclassified, *S. wadsworthensis, A. hadrus, Bifidobacterium* unclassified, *Dorea longicatena, Gemmiger formicillis, Prevotella copri, Roseburia inulinivornas* and *Ruminococcus* 2 unclassified. Two species (*Alistipes putredinis* and *Ruminococcus bromii*) present in the MCC100 were specifically detected in the LS control group. These outcomes suggest MCC100 addition affected the presence of unique species having common and specific effects in CM and LS microbiota types. Common changes were confirmed by analyzing aggregated CM and LS samples (Supplementary file 1 and Supplementary Figure S8a).

In order to study whether the unique species detected by 16S rRNA gene sequencing analysis could be MCC100 members, we compared the unique and shared MCC100 taxa between control and MCC100 supplemented samples in CM and LS groups. Concordant with the previous result, the addition of MCC100 increased the numbers of unique MCC100 taxa in the supplemented CM and LS samples at time 0 and time 3 (Figure 6b). Findings at time 0 are detailed in Supplementary file 1. At time 3, five unique MCC100 strains were identified in the CM microbiota samples supplemented with the MCC100 comprising *B. longum* MCC264 and MCC265 and *Veillonella parvula* MCC755. In the supplemented LS group 13 strains were identified containing *Bifidobacterium adolescentis* MCC257 and MCC258, *P. copri* MCC688, *R. inulinivorans* MCC698 and *S. wadsworthensis* MCC752. Analysis of aggregated CM and LS samples supports identification of *B. longum, B. adolescentis* and *P. copri* MCC strains in supplemented samples after fermentation (Supplementary Figure S8b).

The same species (or species classified up to genus level) to those mentioned above were also identified by 16S rRNA gene sequencing in the previous comparison (Figure 6a), suggesting they could correspond with the MCC100 strains added into the fermentations. These findings indicate that MCC100 supplementation of the elderly microbiota samples introduced taxa that were not present in the original microbiota composition, and several of which were able to integrate into these complex microbial communities under the experimental culture conditions.

### MCC100 supplementation affects the microbiota-associated metabolic profile

**Figure 7.**
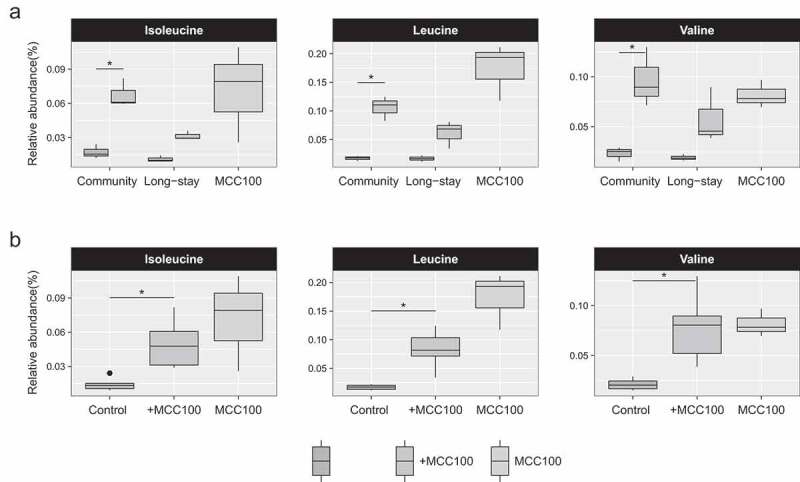
Relative abundance of the branched chain amino acids (BCAA) isoleucine, leucine and valine identified in fermentation samples of the MCC100 (blue), fecal control fermentation samples (red) or fecal control fermentation supplemented with the MCC100 (green) after 3 days of fermentation culture. (a) BCAA in samples separated by microbiota type. (b) BCAA in all aggregated fermenter samples. BCAA were identified by UPLC-MS in both negative and positive ionization modes. Results of negative mode are shown. Statistically significant differences were determined using (a) Mann-Whitney test (*p*-adjust<0.05) and (b) Kruskal–Wallis test (*p*-value<0.05), followed by Dunn’s post-hoc test (*p-value < 0.05)

To explore the effect of MCC100 on the microbial metabolome, we collected the supernatants of the fermentation runs after 3 days and profiled them by untargeted UPLC-MS. In total, 18 and 64 metabolites were identified and annotated by UPLC-MS in negative and positive ionization modes, respectively (Supplementary Table S8). Of these, the relative abundance of the branched chain amino acids (BCAAs) isoleucine, leucine and valine showed an increase in the samples supplemented with the MCC100 compared with controls (Figure 7a). This increment was significant in the CM samples (Ile 0.017% control *vs* 0.068% +MCC100, *p*-adjust = 0.047; Leu 0.017% *vs* 0.106%, *p*-adjust = 0.019; Val 0.023% *vs* 0.097% *p*-adjust = 0.047) and the same trend was observed in the LS group (Ile 0.011% control *vs* 0.032% +MCC100, *p*-adjust = 0.054; Leu 0.017% *vs* 0.061%, *p*-adjust = 0.113; Val 0.019% *vs* 0.058%, *p*-adjust = 0.054). Furthermore, the comparison of the relative abundances of the three BCAAs between control and MCC100-supplemented samples when aggregating CM and LS also retrieved significant differences (Figure 7b and Supplementary Table S8). Association analysis between the relative abundance of the annotated metabolites and the relative abundance of the microbiota species was performed. Positive correlations between the BCAAs and taxa including *B. fragilis, S. wadsworthensis, Veillonella* unclassified, *F. prausnitzii, B. luti, B. pullicaecorum, B. longum, Escherichia/Shigella* unclassified and *C. lactatifermentans* were found (Supplementary Figure S9). Moreover, we confirmed that the MCC100 consortium was able to produce BCAAs by profiling the supernatant of the MCC100 fermentation alone at time 3 (Figure 7, blue bar plots). Then, we explored the potential capability of each MCC100 strain to produce BCAA by *in silico* prediction, revealing that 88 MCC100 strains carried the genes for BCAA biosynthesis in their genomes (data not shown). Of these, MCC100 strains of the same or closely related species (which could have been misclassified by 16S rRNA gene sequencing analysis such as *Blautia* spp., *V. parvula* or *Butyricicoccus faeciformis*) of those that correlated with BCAA production carried the BCAA pathways in their genomes with the exception of *S. wadsworthensis*. These findings suggest the MCC100 strains could be responsible for the measured increase in BCAA levels.

## Discussion

Bacteriotherapies that directly introduce “missing” or depleted taxa could overcome the difficulty of increasing the abundance of desirable species by dietary supplementation.^6^ Although we succeeded in positively modulating taxa and reducing frailty in the recent NuAge study,^23^ this required a year-long total dietary replacement, which is not generally practical. Fecal transplants and artificial bacterial mixture transplants succeed in reestablishing a desirable microbiome configuration in recurrent CDI.^12,15,16^ However, fecal transfers are inefficient in irritable bowel syndrome,^24^ and have low success rates in other conditions such as inflammatory bowel disease.^25^ These findings together with the potential risk of infection by fecal transplants make it an unacceptable option for elder subjects, motivating our current interest in precision microbiota restoration.

The development of synthetic microbial consortia requires first a catalog of gut microbiota members that are generally strictly anaerobes, difficult to isolate and grow. Other researchers made efforts to isolate previously uncultured members of the gastrointestinal tract and increase the number of available genomes.^20–22^ Here, we applied culturomics knowledge to achieve an extensive culture collection from which 100 strains were selected. We performed an intensive characterization including analysis of genes of concern in the 100 genomes and description of their MIC for 7 antimicrobials (discussed in Supplementary file 1) that will allow us to select the safest strains for future use in clinical trials.

Other attempts to re-create artificial stool formulated mixtures with up to 34 different strains selected by different criteria (relatively straightforward to culture or immunomodulation properties).^15,26^ However, the human gastrointestinal tract of an individual harbors around 160 different species^27^ and the gene content of strains from the same species may differ.^28^ The MCC100 contains 100 strains belonging to 75 different species simulating more closely the taxonomic heterogeneity present in the human gut. In fact, bacterial diversity values obtained for MCC100 are in the range of those found in fecal microbiota. Moreover, the MCC100 was formulated with species described as being part of the gut microbiota core and that are associated with diversity and lost in frail elderly.^5^ The MCC100 contained up to 39 taxa absent in the frail microbiota types. The addition of the synthetic consortium affected the diversity of the fermenter ecosystem inoculated with elderly microbiota samples by increasing the alpha-diversity. On the contrary, *in vitro* experiments to asses changes in adult microbiota by dietary fibers retrieved no changes in alpha-diversity.^29^ Systematic review of dietary fiber interventions in healthy adults found no differences with comparators for alpha-diversity,^30^ supporting the hypothesis that bacteriotherapies such as MCC100 supplementation could be more effective for raising microbiota diversity than dietary supplementation.

One of the limitations of this study is that several microbiome differences between different treatment conditions were of borderline statistical significance due to the complexity of microbiota communities together with the inherent limitation of fermenter studies, namely the number of samples and replicates that can be tested. To overcome this, we analyzed the data by comparing MCC100 supplemented and control groups by microbiota-type but also aggregating CM and LS samples together. Moreover, the observed increment of alpha-diversity indices and number of MCC100 taxa being greater in the supplemented samples at inoculation time than final timepoint, indicates a selective effect of the fermentation conditions. The initial loss of diversity is common of *in vitro* gastrointestinal models,^29,31,32^ indicating *in vitro* models have limitations in their ability to mimic human colon conditions and highlighting the importance of confirming the conclusions obtained here in *in vitro* colon models with follow-up experiments in pre-clinical models or human subjects.

To understand what bacterial taxa were responsible for the increase in alpha-diversity after MCC100 addition, we studied changes in relative abundance and the presence of unique species across groups and identified species that were able to integrate in the recipient microbiota communities. MCC100 was able to increase the relative abundance of *B. fragilis, B. luti*, and *S. wadsworthensis* in elderly samples. Notably, according to the iBBiG-defined OTU groups, the first two species were classified in Core and Reduced Core groups and *S. wadsworthensis* in the group associated with health, healthy diet or microbiota diversity^5^ (Supplementary Table S1a). Resonating with this, we previously reported that the *Sutterella* genus is more abundant in CM than LS microbiomes.^3^ MCC100 seemed to prevent the loss due to fermentation of the health-promoting bacterium *F. prausnitzii* in the CM samples but also opportunistic pathogens *Escherichia/Shigella* unclassified in LS samples. This outcome indicates a more nuanced design of the MCC100 is needed for re-programming LS microbiota, and might suggest variable outcomes depending on recipient microbiota as has been observed for fecal transplants.^33^

Similarly, we observed common and different effects of the MCC100 addition on the unique taxa observed for LS and CM samples after fermentation. Supplemented CM microbiotas harbored unique species classified in the Core and Diversity Associated iBBiG-defined OTU groups^5^ (Supplementary Table S1) such as *B. longum, B. pullicaecorum, C. lactatifermentas* and *Veillonella* sp, as well as the health-associated group *Lactobacillus*.^34^ MCC100 addition affected the unique species found in LS microbiota profiles, adding taxa that are classified in the Core (*A. hadrus, Bifidobacterium* sp., *C. lactatifermentans, Ruminococcus* sp, *Veillonella* sp.), and associated with health, healthy diet or microbiota diversity (*B. pullicaecorum, P. copri, R. inulinivorans, S. wadsworthensis*)^5^ and *Lactobacillus* unclassified. Altogether, these findings indicate the positive role of MCC100 in modulating the microbiota of elderly *in vitro*, increasing the alpha-diversity through the addition of previously absent taxa and the modulation of groups that are normally abundant in healthy individuals and that are lost in the transition from CM to LS microbiota profile.

Metabolomic analysis consistently found BCAA increased by the addition of MCC100. This cluster of essential amino acids could have beneficial implications in the health of elderly. Animal models showed that oral BCAA supplementation slows the change speed of gut microbiota,^35^ increased the average life span^36^ and counteracts age-induced sarcopenia in aged rodents.^37^ Oral administration of BCAA has been shown to reduce the incidence of infections acquired in long-term rehabilitation centers.^38^ Furthermore, circulating BCAA are associated with lower dementia and Alzheimer’s disease risk,^39^ and negatively correlate with frailty in long-lived individuals.^40^ Interestingly, BCAAs are under the spotlight since simultaneous studies found association of insulin-resistance in adults with elevated serum BCAA levels and gut microbiota members that have enriched potential for their biosynthesis.^41^ However, other studies indicated the association between serum BCAA and cardiometabolic phenotypes is more pronounced in adults than older subjects, supporting the potential anti-aging effects of the BCAA.^42^ In line with this, low protein intake has been showed to optimize health span in adults aged 65 and younger, but not in older subjects.^43^ Notably, Haran et al. found a reduction of microbiota-BCAA synthesis pathways linked to aging in long-stay residents.^11^ These considerations suggest that the implications of BCAA levels for health are very context dependent but that there is still potential therapeutic value in the appropriate subjects. In light of these results, MCC100 – through the modulation of bacterial BCAA – may exert beneficial physiological roles relevant for elder subjects, particularly those living in nursing homes. Bacteria synthesize BCAA through a conserved pathway.^44^ Accordingly, the majority of MCC100 strains harbored the BCAA synthesis genes. Hence, more studies are needed to clarify the mechanisms behind the alteration of the BCAA metabolism of the microbiota communities by MCC100.

As mentioned above, the constraints of the experiment may be restricting the measurable effect of MCC100 supplementation on the modulation of the microbiota communities and associated metabolome *in vitro*. Thus, although *in vivo* MCC100 engraftment and interaction networks are possibly different, greater changes on recipient microbiota and associated metabolome may be achievable in *in vivo* models. Nevertheless, our *in vitro* findings here must be confirmed in an *in vivo* model that represents better the human gut.

In conclusion, this proof of concept study of a newly isolated culture collection suggests that supplementation with the MCC100 could prevent or correct the gut microbiota alterations associated with unhealthy aging. Further studies are needed to evaluate and confirm the effect of the MCC100 on microbiota programming in *in vivo* models, as well as to describe MCC100 strain compatibility with oral administration. In addition, the well-characterized 100 strains offer the possibility of formulating customized cocktails that treat specific patients with specific microbiota deficiencies. MCC100 could be considered in the future as a live biotherapeutic product for the treatment of diverse conditions where the gut microbiota is altered.

## Materials and Methods

### Fecal sample collection

Donor recruitment was approved by the local Clinical Research Ethics Committee and informed consent was obtained. The volunteers had not received antibiotics for at least 1 month prior to the sampling date (Supplementary Table S9). Fecal samples were transferred to an anaerobic cabinet less than an hour after passing and were manipulated under anaerobic conditions.

### Isolation and identification of microorganisms

Fecal samples were mixed in reduced phosphate buffered saline (PBS), and the homogenate was diluted and spread onto plates of 30 different media (Supplementary Table S10). Plates were incubated under anaerobic conditions in an atmosphere of 90% N_2_, 5% CO_2_, and 5% H_2_ at 37°C for up to 4 days. Isolates were picked and re-streaked to purity. *Methanobrevibacter smithii* was isolated and cultured in Hungate tubes. Pure cultures were stored with glycerol 20% (v/v) at −80°C. DNA was extracted using the Qiagen DNeasy blood and tissue kit (Qiagen). The isolates were identified by PCR amplification of the 16S rRNA gene using the universal bacterial primers 27F2 (AGAGTTTGATYMTGGCTC) and 1492R3 (GGNTACCTTGTTAYGACTT). After Sanger sequencing of the 16S amplicon carried out by GATC Biotech Ltd, reads were aligned in BLASTn^45^ to assign closest species match. Taxonomic assignation of the isolated species in genus, family and phylum was performed with Hierarchy Browser tool of Ribosomal Database Project (RDP).^46^

### Formulation of the MCC100

To select species for assembly into a consortium that would represent the diverse microbiota of a typical healthy subject, publications defining the most prevalent and abundant taxa in the gut were reviewed and cross-referenced against the isolated genera and species (Supplementary Table S1). Additionally, 16S rRNA gene sequences of representative isolates of each species in our collection were compared by BLAST against an in-house iBBiG-OTU database and classified in the iBBiG-defined OTU groups^5^ (Supplementary Table S1a) which define the transition from healthy high-diversity microbiota older subjects to frail low-diversity microbiota type. iBBiG analysis shows that OTUs shared by the core and reduced core groups are some of the most abundant and prevalent and considered particularly important for the normal functions of the microbiota. The database highlights OTUs lost in frail elderly microbiota, associated with health, healthy diet or microbiota diversity and with subjects in long-term residential care. The criteria for species selection were: species or species whose genera have been published as part of the gut microbiota of healthy subjects at least once; species not published as such but classified in the Core, Core-Reduced Core or Diversity Associated iBBiG-OTU groups; other species described as common members of the gut microbiota by other studies, with potential probiotic capability or that may increase the diversity of prevalent genera.

### Genetic dereplication to identify unique microbial isolates

Isolate typing was performed by Random Amplified Polymorphic DNA (RADP-PCR) with the primers M13,^47^ 1254^48^ or OPL5.^49^ At least two primers were used with each isolate. Unweighted Pair Group Method with Arithmetic mean (UPGMA) dendrograms derived from comparison of the RAPD-PCR patterns were performed with BioNumerics software (v 7.6) (Applied Maths) using the Number of Different Bands as similarity coefficient.

### Antimicrobial susceptibility testing

Antimicrobial susceptibility testing was performed following the guidelines and interpretive criteria of the European Committee on Antimicrobial Susceptibility Testing (EUCAST).^50^ The antibiotic test panel was chosen following the recommendations of Brook et al.^51^ for antimicrobials with an available EUCAST breakpoint (Supplementary Table S5a). Antibiotic susceptibility was determined with a minimum inhibitory concentration (MIC) procedure using the E-test gradient strip method (bioMérieux) accordingly to the manufacturer’s instructions. Additionally, strains of enterococci, streptococci and enterobacteria were analyzed for their sensitivity to the antimicrobials and concentrations recommended by EUCAST^50^ and EFSA^52^ with the EUCAST standardized disk diffusion method (Supplementary Table S5c).

### Genome sequencing and analysis

Genome sequencing was performed using the Illumina HiSeqX platform, and 151 bp paired-end reads were generated at BGI (Hong Kong). Briefly, read quality was assessed using FastQC (v0.11.3), genome assembly was performed using Velvet (v1.2.10) as previously described,^53^ and annotation using RAST tool kit (RASTtk).^54^ Genome analysis is detailed in Supplementary file 1. Genomes were classified within the “Most wanted” taxa for genome sequencing priority list of the Human Microbiome Project (HMP) (http://hmpdacc.org/most_wanted/#data). Strains with 100% identity over the full length of the partial 16S rRNA gene sequence provided by HMP were considered as “Most Wanted” taxa.

### Taxonomic classification and phylogenetic analysis

Full-length 16S rRNA genes were reconstructed from genome assemblies and subjected to nucleotide BLAST (BLASTn) against the NCBI Nucleotide Collection (nr) with exclusion of uncultured/environmental sample sequences (accessed December 2018). Selected strains with BLASTn ambiguous results were further classified (Supplementary file 1). Taxonomic classification from phylum to genus was retrieved from the RDP using the Hierarchy Browser (accessed December 2018). For ambiguous strains (e.g. unclassified at species level), taxonomic hierarchy was confirmed using the RDP classifier against the 16S rRNA training set number 16.^55^ Multiple sequence alignments on 16S rRNA genes were carried out using Infernal 1.1^56^ and the covariance model “SSU_rRNA_bacteria” (RF00177, Rfam). Low-confidence regions of alignments were removed with gblocks 0.91b^57^ using default parameters except for “minimum length of a block” set to 5, as recommended for rRNA genes. Maximum likelihood trees were calculated from the trimmed alignments using the Generalized Time-Reversible model with CAT approximation with 20 rate categories in FastTree2.^58^ Resulting trees were rooted, formatted and grouped in MEGA-X.^59^

### Preparation of MCC100 suspensions and fecal inocula

The selected strains were grown individually in their routine media. After 24 h the OD_600_ was measured and different volumes of the 99 bacterial strains were mixed up to 1 L total volume (Supplementary Table S3). Volumes were adjusted to achieve end-proportions based upon the described abundances of the respective microbial groups in the human gut microbiota (references in Supplementary Table S1b). OD values were taken into account and additional adjustment was made to maintain the proportions. The mixture was centrifuged at 5000 rpm at 4°C for 10 min. The pellet was resuspended in ½ volume of reduced PBS and glycerol (20% v/v) and 20 mL aliquots were stored at −80°C until use. All the work was performed under anaerobic atmosphere except the centrifugation step. The archaeal strain *Methanobrevibacter smithii* MCC662 was grown for 1 week and 0.25 mL aliquots of culture mixed with glycerol 20% (v/v) were stored frozen. Each fecal sample was homogenized with reduced PBS at 20% (w/v) into a stomacher bag with a 70 µm filter insert (Sparks lab supplies). The filtered fecal suspension was adjusted with glycerol to a final concentration of 20% (v/v).^60^ Aliquots were stored frozen at −80°C.

### *In vitro* fermentations

Human colon conditions were simulated in a single-stage continuous fermentation system (MiniBio Reactors, Applikon Biotechnology) as described elsewhere.^31^ Temperature was controlled at 37°C, pH at 6.8 and stirrer at 100 rpm. Anaerobic conditions were automatically maintained by supply of O2-free N2 gas. Cultures were run for 3 days in continuous flow, with 24 h retention time. Fermentations were performed in 400 mL working volume with fermentation medium described in Supplementary Table S10 (medium 32). Fecal and MCC100 inoculum aliquots were thawed in an anaerobic cabinet for 30 min. Two vessels were inoculated in parallel with the same fecal sample at 1% (w/v) and one vessel received the MCC100 consortium while the other was the control condition. The MCC100 inoculum was added at 4 × 10^6^ cfu mL^−1^ final concentration. Samples were collected at time 0 and after 3 days of culture (time 3) in continuous flow. Samples were centrifuged, and pellets and filtered supernatants were kept at −80°C for further analysis. The experiment was run in duplicate for each fecal sample. Fermentations of the MCC100 alone were performed in triplicate.

### 16S rRNA gene amplicon sequencing and microbiota composition analysis

DNA was obtained with the QIamp Fast DNA Stool kit (Qiagen). The V3-V4 region of the 16S rRNA gene was amplified, sequenced and analyzed as described by Ribiere et al.^61^ (Supplementary file 1). BLAST (blastn) searches comparing the 16S rRNA gene full-length sequences of MCC100 strains to V3/V4 reads from the 16S rRNA gene sequencing dataset (after quality filtering), were used to determine the presence of MCC100 taxa in the fecal and fermenter samples. BLAST results were filtered at 98.7% identity and 90% coverage of the query gene. Allowing for potential sequence errors, the 98.7% cutoff falls between species- and strain-level assignment. The number of BLAST hits for MCC100 strains 16S rRNA gene sequences was counted per sample, with one hit or greater interpreted as the presence of that strain.

### Metabolomic profiling

Untargeted metabolomics of the supernatants recovered from fermentation were performed by ultra-performance liquid chromatography (UPLC) coupled with a quadrupole-Time of Flight Mass Spectrometer (q-TOF-MS)^62^ (Supplementary file 1). Valine, leucine and isoleucine were confirmed by authentic standards obtained from Sigma Aldrich.

### Statistical analysis

R statistical software package (version 3.4.3) was used for statistical analysis and data visualization (ggplot2 2.2.1). Principal coordinates analyses (PCoA) (made4 1.50.1) were performed on unweighted and weighted UNIFRAC distances matrices and differences between groups were tested using ANOSIM (Analysis of similarities) (vegan 2.4–3). Significant variations in alpha diversity, taxa abundance and metabolites abundance were assessed using Mann-Whitney test for paired and unpaired data. When comparing more than two experimental groups, the Kruskal–Wallis test was used followed by Dunn’s test for post-hoc analysis (R package dunn.test v1.3.4). *P*-values were adjusted for multiple testing using Benjamini-Hochberg correction. Correlations between metabolite and taxa relative abundances were calculated using standard Spearman’s rank correlation and hierarchical clustering was computed using the hclust function in R (method “complete”).

## Data availability

All 16S rRNA gene sequence data are available through the Sequence Read Archive (SRA) under accession numbers listed in Supplementary Table S11. The genome sequences of the 100 strains have been deposited in NCBI under the project number PRJNA548918 with individual accession numbers listed in Supplementary Table S4.

## Supplementary Material

Supplemental Material
