# Supplementary material for: A synthetic consortium of 100 gut commensals modulates the composition and function in a colon model of the microbiome of elderly subjects
Source: Gut Microbes. 2021 May 16;13(1):1919464. doi: 10.1080/19490976.2021.1919464 (PMC8128205; doi:10.1080/19490976.2021.1919464)
Supplement: Supplemental Material [file KGMI_A_1919464_SM3620.zip › Supplementary information/Supplementary Figures_REVISED.pdf]

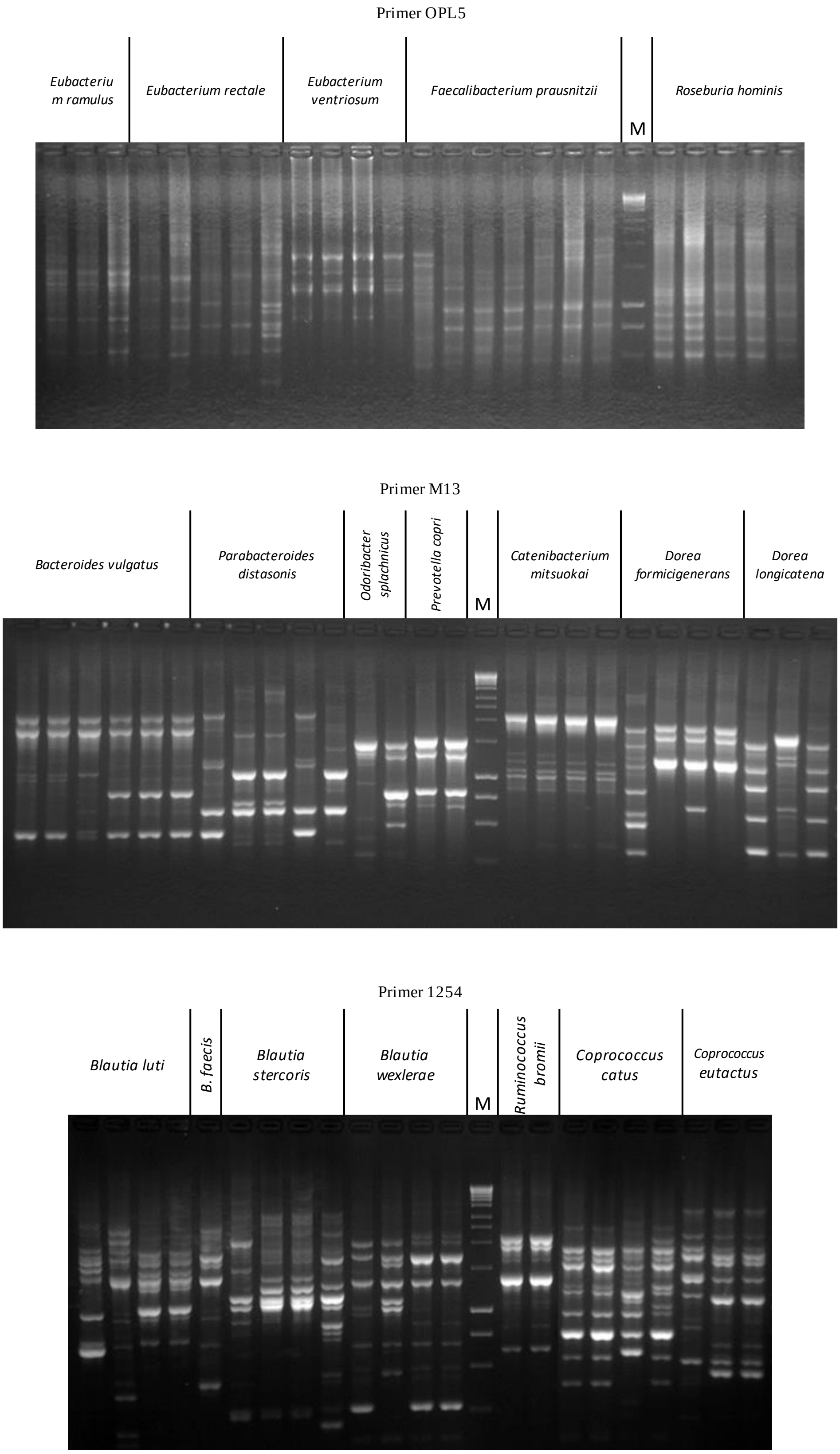

Supplementary Figure S1: Example of RAPD-PCR profiles obtained with the primers OPL5, M13 and 1254. Each lane corresponds to an isolate of the indicated closest species. Lane M: 1-kb DNA molecular mass ladder (Bioline).

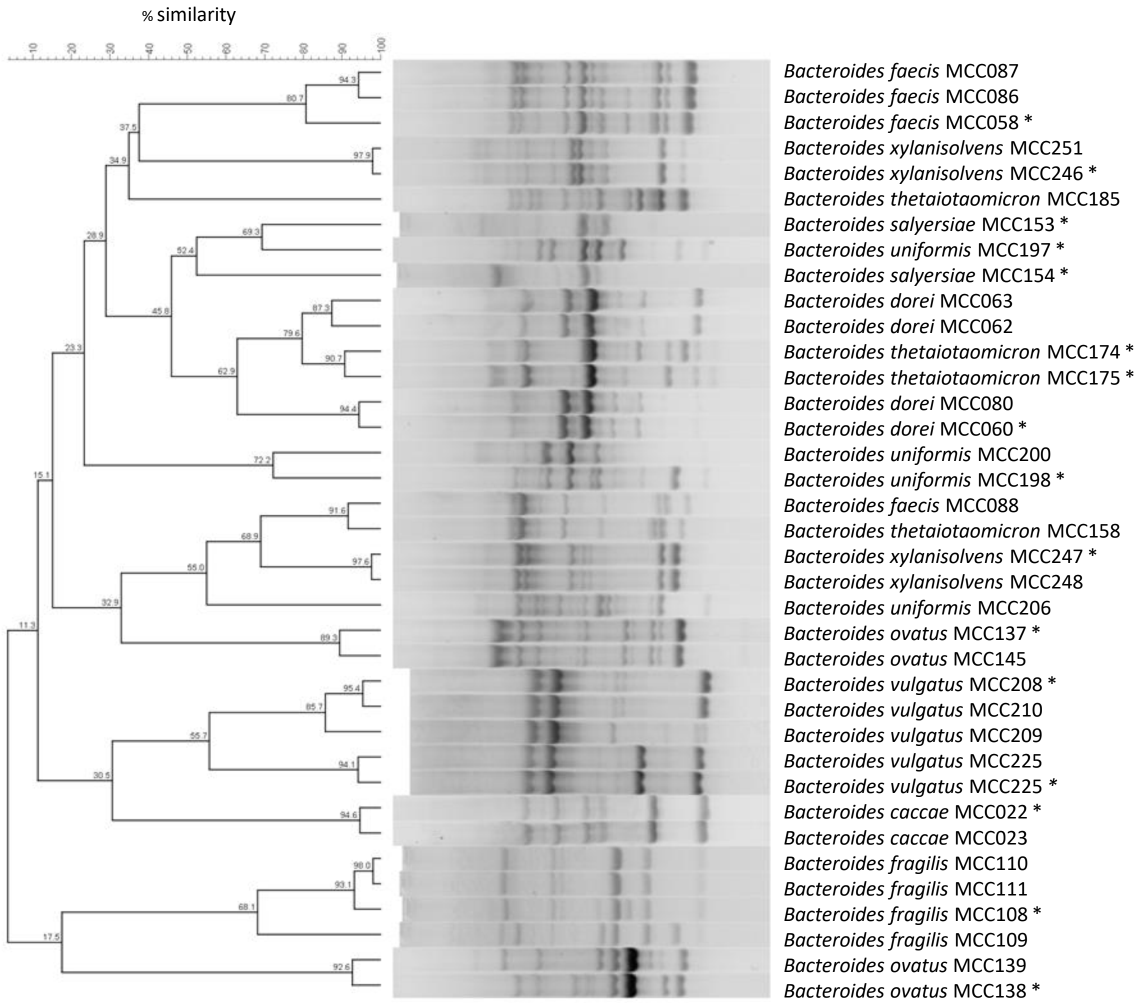

Supplementary Figure S2: Example of an UPGMA dendrogram derived from a comparison of the RAPD-PCR patterns obtained with primer M13 for the isolates of the genus *Bacteroides*. Dendrograms were performed using Number of different bands as similarity coefficient. Asterisks indicate the strains selected for the artificial consortium MCC100.

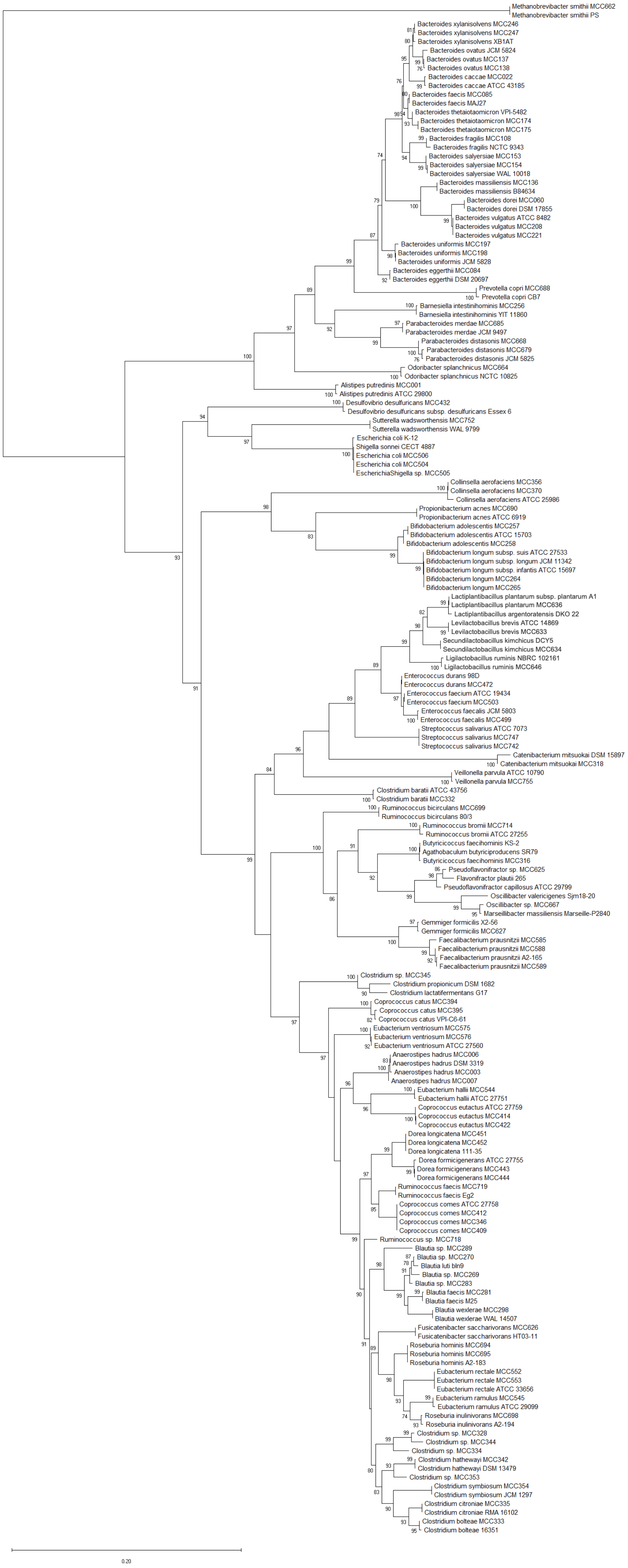

Supplementary Figure S3: Phylogenetic positioning of the MCC100 strains among 74 reference strains. Phylogenetic maximum likelihood tree using the Generalized Time-Reversible model with CAT approximation with 20 rate categories inferred from the 16S rRNA gene. The tree is rooted on the domain Archaea for illustrative purposes. Local support values superior or equal to 70% are displayed.

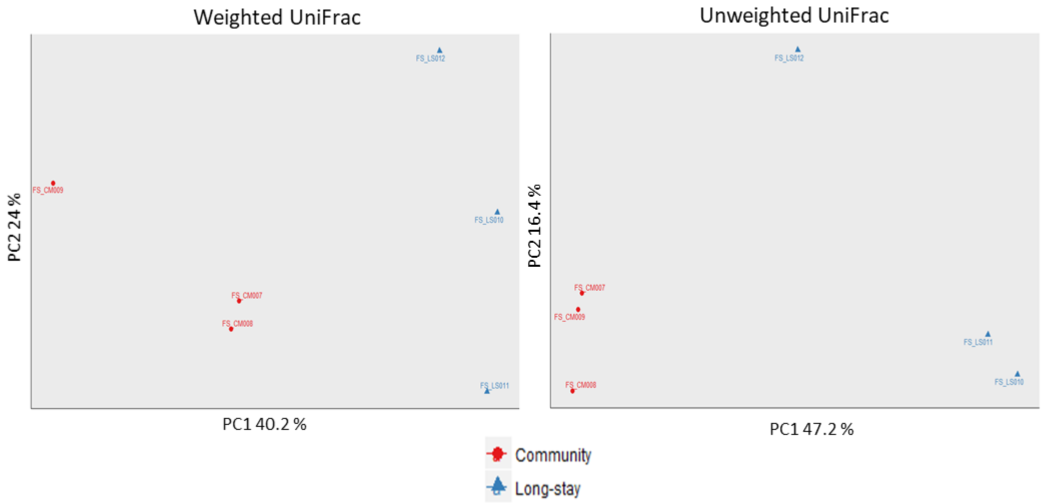

Supplementary Figure S4: Principal coordinate analysis (PCoA) differentiates microbiota patterns of elderly donors living in the community (red) and long-stay care (blue) on weighted (left) and unweighted (right) UniFrac distance matrices.

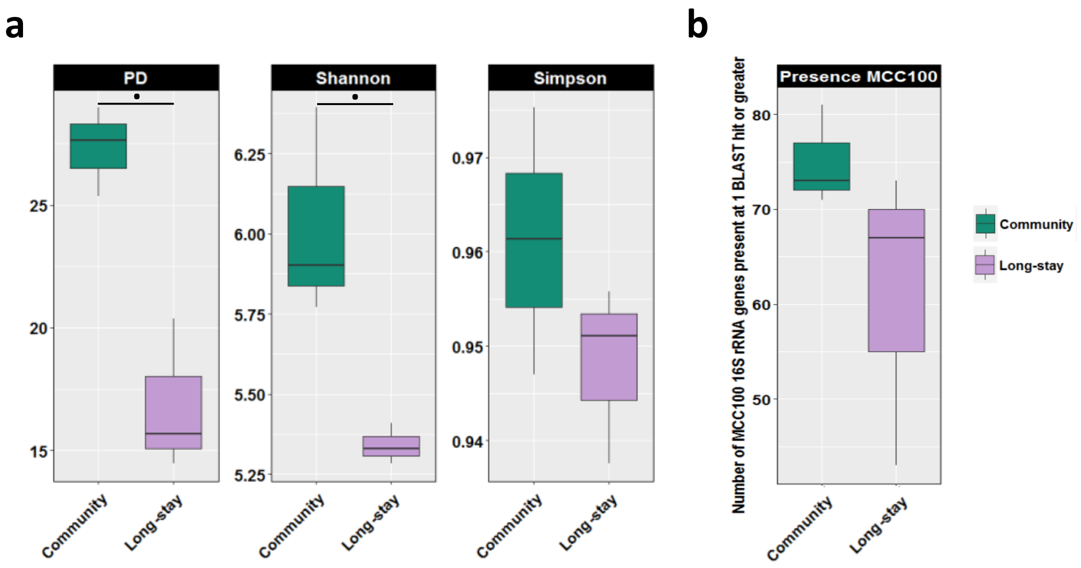

Supplementary Figure S5: (a) Alpha-diversity indexes of the gut microbiota of elderly donors living in the community and long-stay care. (b) Presence of MCC100 species across donors microbiota. BLAST results of the MCC100 16S rRNA gene full-length sequences against the V3/V4 16S rRNA gene sequences of the faecal samples were filtered at 98.7% identity and 90% coverage. MCC100 taxa with one hit or greater were considered as present in the sample. Statistically significant differences were determined using Mann-Whitney (one-tailed) (• exact p-value=0.05).

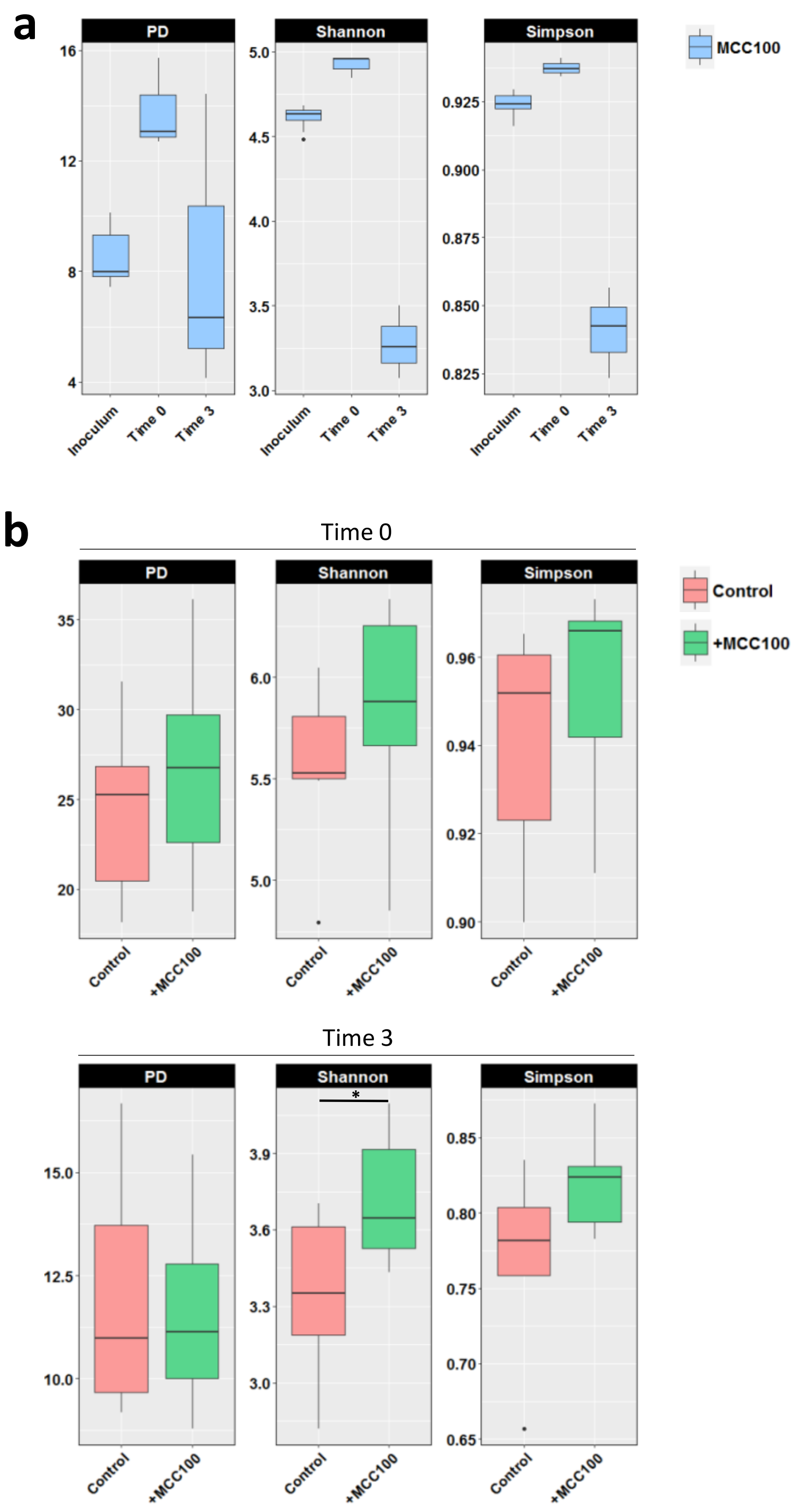

Supplementary Figure S6: Alpha-diversity indexes of (a) synthetic consortium MCC100 inoculum and fermentation samples of the MCC100 alone (time 0 and time 3); and (b) aggregated faecal fermentations supplemented with MCC100 (green) or control (red) at time 0 and after 3 days of culture (time 3). Statistically significant differences between control and MCC100 supplemented fermentation groups were determined using Mann-Whitney (one-tailed) (* p-value < 0.05).

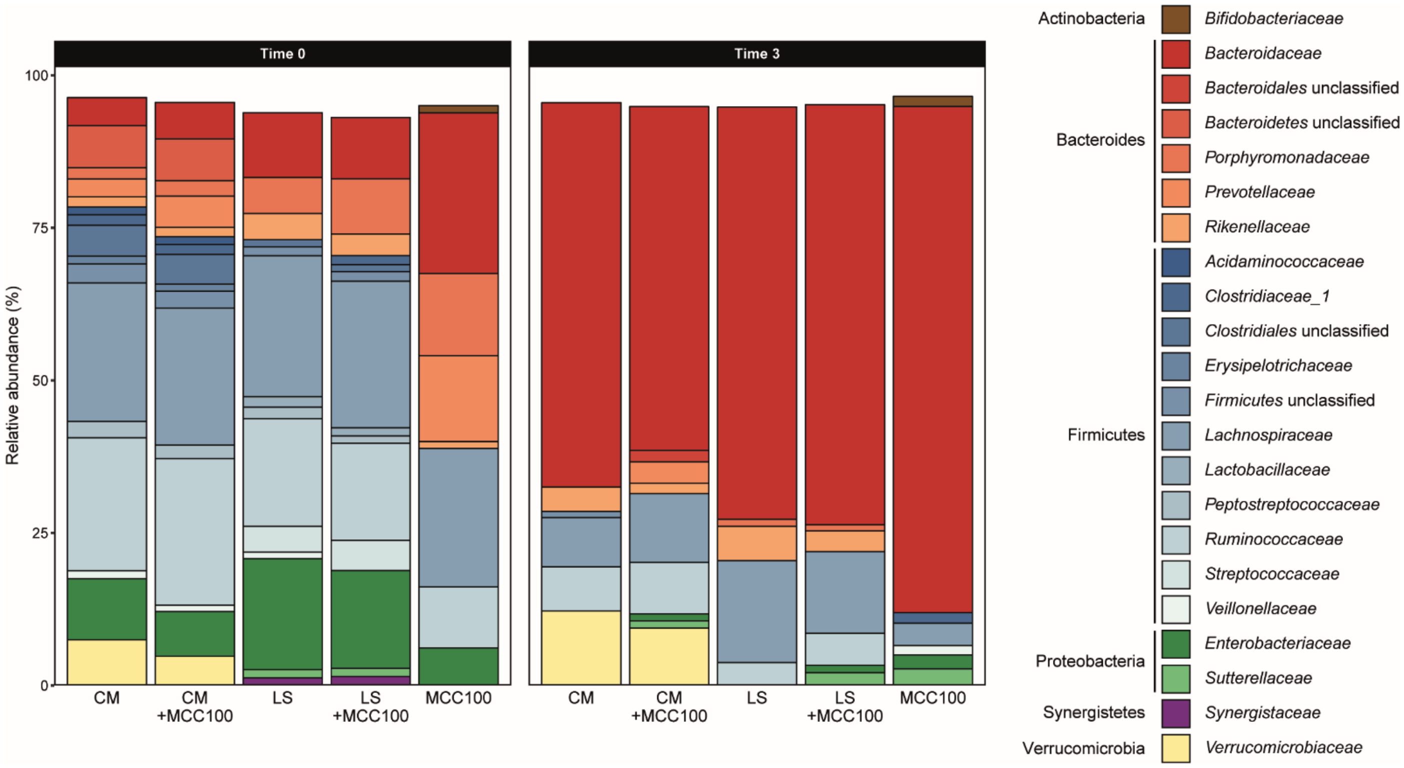

Supplementary Figure S7: Bacterial community profiles at family level for control and MCC100 supplemented microbiota typesand MCC100 fermentation alone at time 0 and time 3. Only dominant bacterial species (relative abundance ≥ 1%) are displayed. Species within the same phylum are indicated by different shades of the same colour.

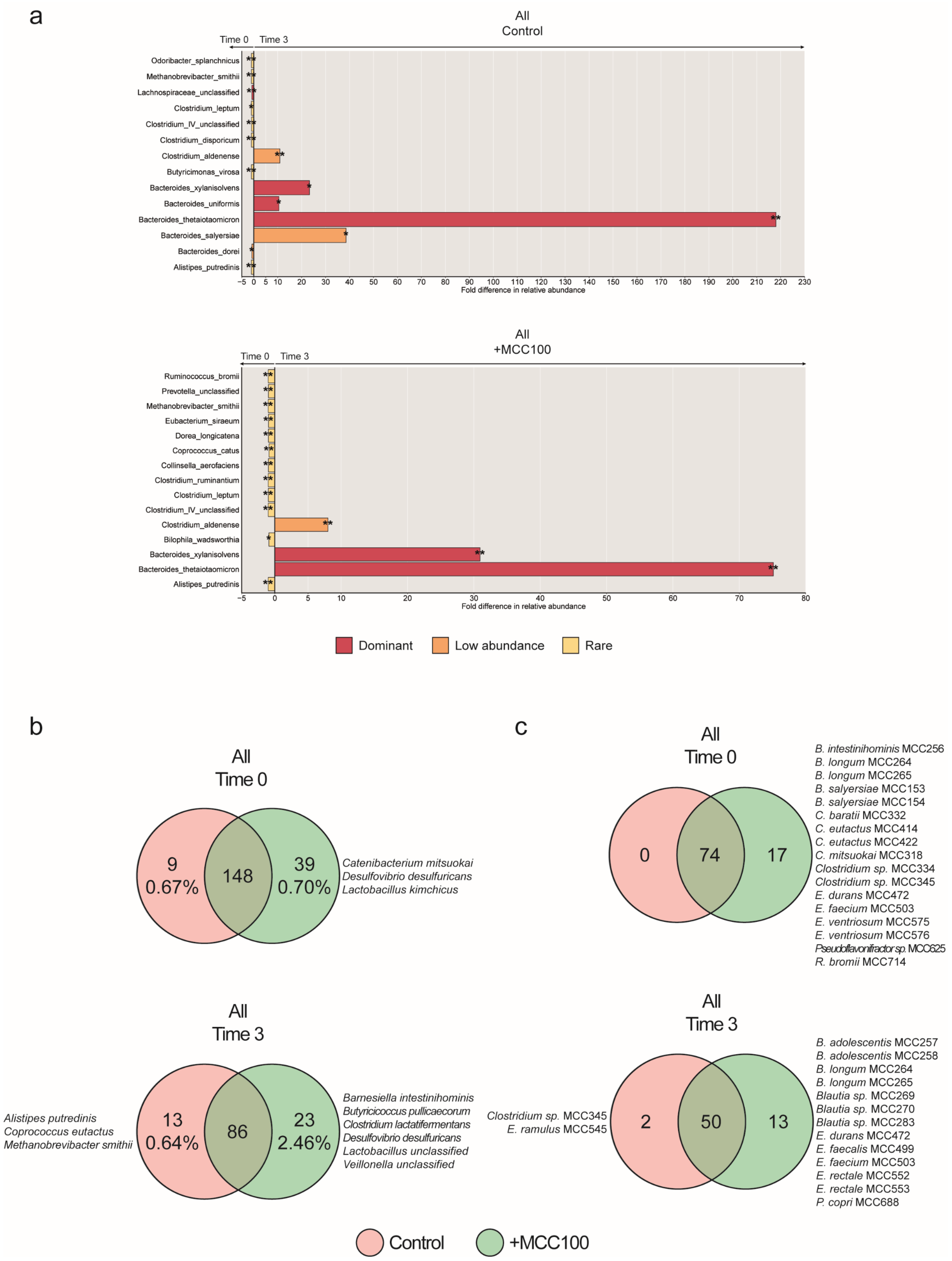

Supplementary Figure S8: (a)Fold differences in relative abundance of bacterial species showing significant differences between time 0 and time 3 for control and MCC100 supplemented fermentations. Bacterial species were classified as dominant (relative abundance ≥ 1%), low abundant (relative abundance between 0.1 and 1%) or rare (relative abundance ≤ 0.1%). Statistically significant differences were determined using Kruskal-Wallis (p-adjust<0.01) + Dunn's test (* $p<0.05$ ** $p<0.005$). (b) Shared and unique bacterial species detected in 16S rRNA gene sequencing analysis at time 0 and time 3 in MCC100 supplemented (green) and control (red) fermentations (species that were present in both technical replicates and in at least 50% of the samples in each group). (c) MCC100 taxa identified in MCC100 supplemented and control fermentations at time 0 and time 3 by BLAST searches of the MCC100 16S rRNA gene full-length sequences against the V3/V4 16S rRNA gene sequences.

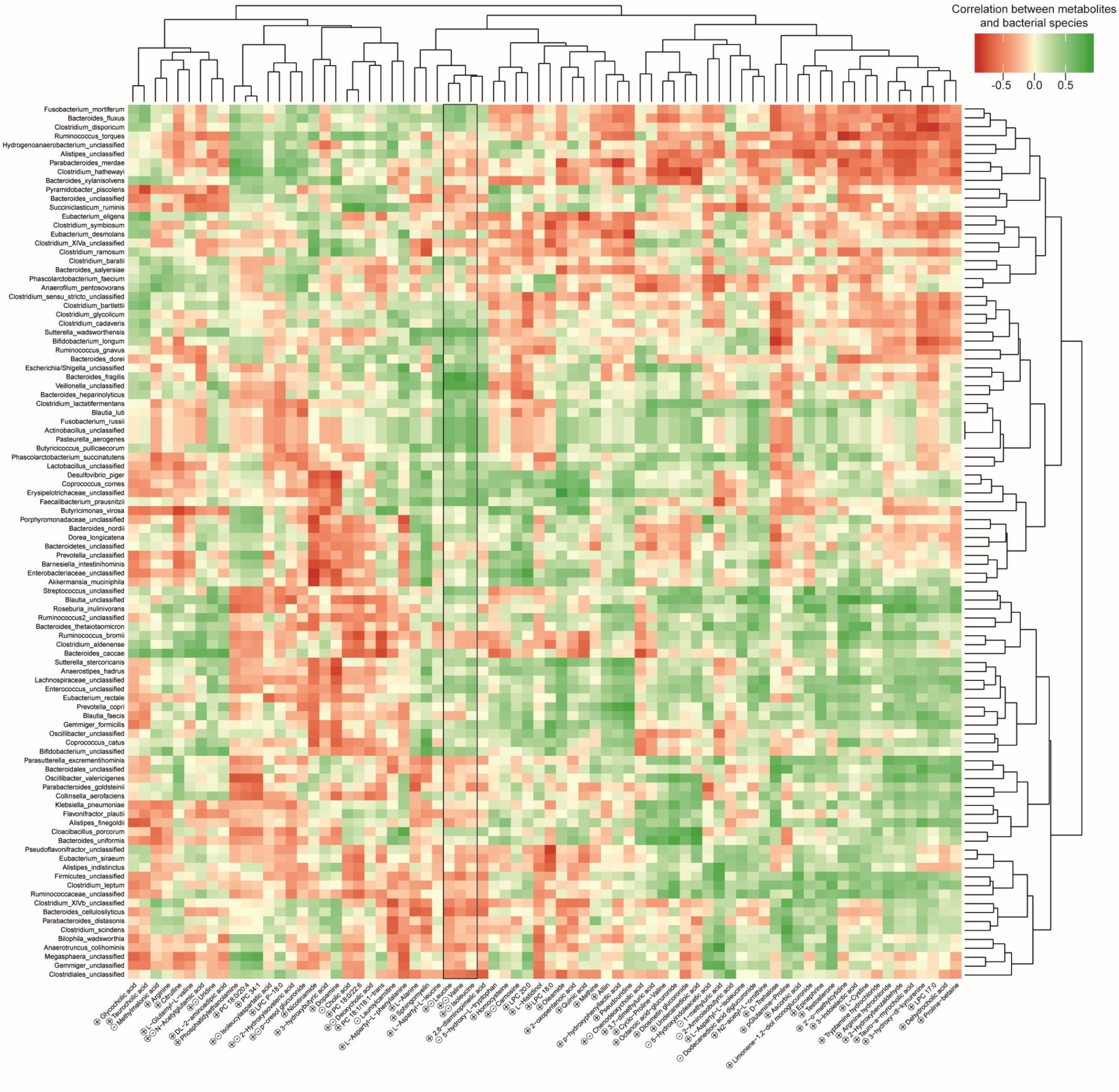

Abbreviations	
LPC 20:0	1-Eicosanoyl-sn-glycero-3-phosphocholine
LPC P-18:0	1-1Z-Octadecenyl-sn-glycero-3-phosphocholine
LPC 17:0	1-heptadecanoyl-2-hydroxy-sn-glycero-3-phosphocholine
LPC 18:0	1-stearoyl-2-hydroxy-sn-glycero-3-phosphocholine
PC 18:0/20:4	1-stearoyl-2-arachidonoyl-sn-glycero-3-phosphocholine
PC 18:0/22:6	1-stearoyl-2-docosahexaenoyl-sn-glycero-3-phosphocholine
PC 34:1	1,2-diacyl-sn-glycero-3-phosphocholine
PC 18:1/18:1-trans	2-1Z,9Z-1,9-Octadecadien-1-yloxy-3-11Z-11-octadecenoyloxypropyl 2-trimethylammonioethyl phosphate

Supplementary Figure S9: Association between the relative abundance of annotated metabolites and the relative abundance of bacterial species. Spearman's rank correlations were calculated between the relative abundance of metabolites putatively identified by UPLC-MS (negative (-) and positive (+) ionization modes) and relative abundance of bacterial species in control and MCC100 supplemented fermentations at time 3. Unsupervised hierarchical clustering was applied on metabolites and bacterial species. The BCAA leucine, valine and isoleucine are indicated in a square.
