# Supplementary material for: A synthetic consortium of 100 gut commensals modulates the composition and function in a colon model of the microbiome of elderly subjects
Source: Gut Microbes. 2021 May 16;13(1):1919464. doi: 10.1080/19490976.2021.1919464 (PMC8128205; doi:10.1080/19490976.2021.1919464)
Supplement: Supplemental Material [file KGMI_A_1919464_SM3620.zip › Supplementary information/Supplementary file 1_REVISED.docx]

Supplementary file 1

A synthetic consortium of 100 gut commensals modulates the composition and function in a colon model of the microbiome of elderly subjects

Marta Perez^1^, Alexandra Ntemiri^1^, Huizi Tan^1^, Hugh M. B. Harris^1^, Henrik Munch Roager^2^, Lars Ove Dragsted^2^, Celine Ribiere^1^, Paul W. O’Toole^1^

^1^ School of Microbiology and APC Microbiome Ireland, University College Cork, Cork, Ireland

^2^Department of Nutrition, Exercise and Sports, University of Copenhagen, Frederiksberg, Denmark

Correspondence to: Prof. Paul W. O’Toole, School of Microbiology & APC Microbiome Ireland, Food Science Building, University College Cork, T12 YN60 Cork, Ireland; Tel: +353 21 490 3997; email: [pwotoole@ucc.ie](mailto:pwotoole@ucc.ie)

# Supplementary Results

## Establishment of the Microbiome Culture Collection (MCC)

Thirty different culture media (Supplementary Table S10) were used to anaerobically isolate commensal microbes from the faecal samples of seven healthy donors (Supplementary Table S9). Purified isolates were identified to the closest species by 16S rRNA gene amplicon sequencing analysis. The isolates belonged to 86 bacterial species from 40 different genera and 4 bacterial phyla, and one archaeon species (Supplementary Table S1a).

## Changes in microbiota composition and taxa relative abundance in elderly microbiota types supplemented with the MCC100 in a colon model

PCoA on unweighted and weighted UNIFRAC distance matrices were constructed to assess for differences in microbiota composition and relative abundance between CM and LS fermentations with and without the MCC100 supplementation (Supplementary Figure S6). At time 0, based both on unweighted and weighted UNIFRAC distance matrices, CM and LS samples were significantly separated whether they were supplemented with the MCC100 (ANOSIM p-values of 0.002 and 0.005, for unweighted and weighted UNIFRAC, respectively) or not (ANOSIM p-values of 0.001 and 0.007, for unweighted and weighted UNIFRAC, respectively). This showed that CM and LS samples differed both in taxon composition and relative abundance and that microbiota type is the main driver of variability between the samples at time 0, whether the samples were MCC100 supplemented (ANOSIM R values of 0.835 and 0.441, for unweighted and weighted UNIFRAC, respectively) or not (ANOSIM R values of 0.939 and 0.554, for unweighted and weighted UNIFRAC, respectively). No significant separation was observed between CM and LS microbiotas with MCC100 supplementation compared with their corresponding control groups. At time 3, PCoA analysis on unweighted and weighted UNIFRAC distance matrices showed the same separation profiles as at time 0 with microbiota type as the main driver of variability between samples. However, the proportion of variability explained by microbiota type was lower at time 3 compared to time 0 (ANOSIM R values of 0.613 vs 0.939 and 0.174 vs 0.554, for unweighted and weighted UNIFRAC, respectively), suggesting a selective effect from the fermentation process both on microbiota composition and relative abundance. Moreover, R values derived from ANOSIM tests showed that a lower proportion of the variability between CM and LS samples was explained when supplemented with the MCC100 compared to the control groups (ANOSIM R value of 0.370 vs 0.613 for unweighted UNIFRAC), suggesting that upon receiving the MCC100 supplementation, CM and LS samples are more similar in composition than their control groups at time 3.

The differences in relative abundance of the microbiota species between time 0 and time 3 in CM and LS samples with the MCC100 and control was studied. Fermentation caused changes in the relative abundance of taxa in both the MCC100 supplemented and control CM groups including reductions of *F. prausnitzii*, *Eubacterium desmolans*, *Clostridium disporicum* and *Anaerostipes hadrus*, and an increase in *Clostridium aldenense* proportions. Likewise, reductions of unclassified *Escherichia/Shigella* and a rise in *Bacteroides thetaiotaomicron* were observed between time 0 and time 3in the LS samples with and without the consortium addition.

Comparisons of time 0 and time 3 after aggregating datasets across CM and LS samples but keeping them separated by supplementation condition revealed significant increments in *B. thetaiotaomicron* and *Clostridium aldenense* (Supplementary Figure S8a).

Comparisons of unique and shared species between control and MCC100 supplemented fermenter data after aggregating CM and LS samples was performed. 23 species were specifically detected in the MCC100 supplemented group at the final time point, including *B. pullicaecorum*, *C. lactatifermentans*, *Veillonella* unclassified, *Lactobacillus* unclassified and *Desulfovibrio desulfuricans* (Supplementary Figure S8b). *C. eutactus*, *M. smithii* and *A. putredinis* were among the taxa only detected in the control group.

MCC100 supplementation increased the number of unique MCC100 taxa in all conditions (Figure 5b). Unique MCC100 taxa were detected at time 0 in CM+MCC100 samples including *B. longum* MCC264 and MCC265 (also identified at time 3), *Catenibacterium mitsuokai* MCC318 and *Clostridium symbiosum* MCC354. *C. eutactus* MCC414 and MCC422 and *M. smithii* MCC662 were exclusive of the CM control group at time 3 among a total of 6 MCC100 strains. In LS+MCC100 group 26 MCC100 unique strains were specifically detected at time 0 including *C. mitsuokai* MCC318, *C. eutactus* MCC414 and MCC422, *D. desulfuricans* MCC432, *Enterococcus faecalis* MCC499 and *S. wadsworthensis* MCC752 (which was also detected at time 3). LS control samples exclusively contained *A. putredinis* MCC001.

Moreover, the comparison between unique and shared MCC100 taxa after aggregating CM and LS samples showed 17 strains specifically detected in the supplemented group at time 0 consisting *B. longum* MCC264 and MCC265, *C. mitsuokai* MCC318 and *C. eutactus* MCC414 and MCC422. At time 3, 13 unique strains were identified in this condition including *B. longum* MCC264 and MCC265, *B. adolescentis* MCC257 and MCC258 and *P. copri* MCC688 (Supplementary Figure S8c).

# Supplementary discussion

## Antimicrobial susceptibility of the MCC100 bacterial strains

The antimicrobial susceptibility testing methods has not yet been established for anaerobes, therefore, a MIC procedure is recommended by the responsible agencies. Here, we studied the reported the MIC values to 7 antibiotics of 99 bacterial MCC strain. *Bacteroides* spp. and *Parabacteroides* spp. Strains were resistant to benzylpenicillin presumably due to production of beta-lactamase. The common mechanism of resistance has not been shown to be transferable.^1^ Benzylpenicillin resistance is inherent to enterobacteria^2^ and could be intrinsic in *Enterococcus* spp.^3^ Low sensitivity to penicillin has been found among many gut micoorganisms such as *Prevotella* spp.,^4^ *Desulfovibrio* spp.,^5^ *Clostridium* spp.,^6^ *Lactobacillus* spp.^7^ or *Collinsella* spp.^4^ However, the mixture of a penicillin and a beta-lactamase inhibitor is proven to be very efficient against the vast majority of anaerobes.^6^ Accordingly, all the strains tested were sensitive to amoxicillin-clavulanate. Only one strain showed intermediate resistance to imipenem. It possess excellent activity against aerobic bacteria as other carbapenems^8^ Although resistance to chloramphenicol is rare, it has been previously described for some *Bacteroides* spp.^6^ as well as *D. desulfuricans.*^9^ However, no *Collinsella* spp. strain has been reported as chloramphenicol resistant so far. Clindamycin resistances have been previously reported in *Bacteroides* spp., *Clostridium* spp., *Eubacterium* spp. and *Lactobacillus* spp. among others^4,6,7^ and have showed to be intrinsic in *Enterococcus faecalis*^3^ and enterobacteria.^2^ Metronidazole is frequently used for treatment of anaerobic infections of the digestive tract. However, this drug is ineffective against aerobic cells since they lack the electron-transport proteins with sufficient negative redox potential to reduce metronidazole to the active form.^10^ In accordance to our results, the resistance of all lactobacilli to this antibiotic has been repeatedly reported.^7,11^ Similarly, *Propionibacterium acnes* is uniformly metronidazole resistant.^6^ The variable sensitivity to this drug has been indicated in bifidobacterial strains since some of the lack the electron-transport proteins,^7,12^ accordingly 3 out of 4 strains analyzed here were resistant. Therefore, all the metronidazole resistant strains detected here would be intrinsically resistant. Vancomycin intrinsic resistance in lactobacilli is also wall documented and the high level resistance four in the 4 *Lactobacillus* spp. strains is in accordance with previous observations.^7,11^ Thus, only one out of 67 Gram-positive anaerobes (*Clostridium baratii* strain) was resistant to vancomycin being compatible with a transmissible determinant.

# Supplementary Materials and Methods

## Genome sequencing and analysis

Read quality was assessed using FastQC (v0.11.3) and reads were assembled using Velvet (v1.2.10) ^13^ with kmer length = 91 and coverage parameters (exp_cov and cov_cutoff) set to ‘auto. Assembly statistics and quality checks were performed as indicated by Harris et al^14^ (Supplementary Table S4). Additionally, genomes were compared by BLAST (BLASTn v2.2.26+)^15^ against the RDP database (downloaded 05/08/2018)^16^ with only complete or near-complete 16S rRNA genes annotated to species level to confirm no other good hit (>1000 bp; identity >= 97%) was found, which would indicate possible contamination. The genes comprising the Branch Chain Amino Acid (BCAA) pathways^17^ were manually collated in RASTtk annotation results to predict the potential metabolic capabilities of the strains to produce isoleucine, leucine and valine. Putative antibiotic resistance genes and virulence factors were predicted by respectively BLAST comparing amino acid sequences against the Comprehensive Antibiotic Resistance Database (CARD, downloaded 19/02/2019)^18^ and the core dataset of the virulence factor database (VFDB, downloaded 19/02/2019)^19^ with ≥70% identity and ≥70% coverage length^20^. Genetic loci for bacteriocin production were predicted using BAGEL4 (Van Heel et al. 2018) and BLAST results were filtered by ≥40% identity, ≥70% query coverage, ≥70% reference gene coverage and e-value ≤ 1.

## Taxonomic classification for ambiguous strains

Additional phylogenetic analyses on 16S rRNA genes were carried out to further classified ambiguous strains (*i.e.* sequence identity < 98.5% or sharing 99% of sequence identity with more than one species). MCC strains and previously published 16S rRNA sequences were grouped at genus or family level as follow: MCC667 in *Oscillibacter* group^21^; MCC755 in *Veillonella* MCC328^22^; MCC316 in *Butyricicoccus* group^23,24^; MCC328, 334, 344, 345 and 353 in C*lostridium* group^25–27^; MCC269, 270, 281, 283, 289, 298, 443, 444, 544, 718 and 719 in *Lachnospiraceae* group (with exclusion of *Clostridium*)^28–30^; MCC625 in *Pseudoflavonifractor/Flavonifractor* group^31^; MCC085, 246, and 247 in *Bacteroides* group^32^; MCC472 in *Enterococcus* group.^33^ MCC755 taxonomic classification as *Veillonella parvula* was confirmed using *rpoB* gene for blastn searches.^22^ Classification at species level of MCC742 belonging to *Streptococcus* was carried out using blastn with the manganese-dependant superoxide dismutase gene (*sodA*).^34^ Finally, MCC504, 505 and 506 were classified within the Escherichia/Shigella group using a maximum likelihood tree inferred from the concatenation of 3 genes: *gryB*, *mdh* and *rpoB.*^35–37^

## 16S rRNA gene amplicon sequencing and microbiota composition analysis

DNA from pellets of 2 mL fermentation samples, 0.2 mL faecal inocula, 0.2 mL MCC100 inoculum and 0.2 g of faecal samples was extracted using the QIamp Fast DNA Stool kit (Qiagen) as performed previously.^39^ V3-V4 region amplification was performed with the primers S-D-Bact-0341-b-S-17 and S-D-Bact-0785-a-A-21.^40^ The sequencing library was prepared using Nextera XT V.2 Index Kit (Sets A and D, Illumina) according to the Illumina 16S Metagenomic Sequencing Library protocol. PCR products were purified with the SPRIselect reagent kit (Beckman Coulter). Amplicons were quantified with a Qubit dsDNA HS Assay Kit (Thermo Fisher Scientific) and pooled at the same concentration. Sequencing was performed on an Illumina MiSeq Platform (2x250 bp reads) by the Teagasc Next Generation DNA Sequencing Facility (Fermoy, Ireland).

V3/V4 paired-end reads were joined using FLASH programme.^41^ QIIME’s split_libraries_fastq.py was used for quality filtering.^42^ Primers were removed with cutadapt tool^43^ and QIIME’s script truncate_reverse_primer.py. Sequences were filtered by length, sorted by size, single unique reads removed, and the remaining reads were clustered into OTUs (97% of identity) using the USEARCH sequence analysis tool.^44^ Chimeras were removed with UCHIME, using the GOLD reference database. The original quality filtered sequences (6773414 sequences) were mapped onto the 1 821 OTUs with 97% similarity, with 7 734 to 64 623 sequences mapped per sample (on average, 33 340 sequences per sample). OTU representative sequences were classified with 80% confidence threshold from phylum to genus level by MOTHUR^45^ using the RDP reference database and to species level using SPINGO^46^ (Supplementary Table S12). Alpha and beta diversity analyses were conducted using QIIME on a rarefied OTU table to 7 734 reads.

## Metabolomic profiling

Supernatants recovered from fermentation samples were subjected to untargeted metabolomic analysis. 10 µL of each sample was mixed with 10 µL internal standard (Glycocholic acid (Glycine-1-13C), Lysophosphatidylcholine (17:0), Arginine (U-13C6), Adenine (8-13C), L-Tryptophan (15N2), Uric acid (1,3-15N2) and Hippuric acid (1-13C)) and 200 µL MeOH and left at 4°C for 5 min. The mixture was centrifuged at 14.000 rpm at 4 °C for 5 min and the supernatant was transferred to a 96-wells plate. The plate was dried under vacuum and the samples were reconstituted in 200 µL 0.1% formic acid in Mili-Q water. The samples were randomised and analysed by ultra-performance liquid chromatography (UPLC) coupled with a quadrupole-Time of Flight Mass Spectrometer (q-TOF-MS) equipped with an electrospray ionization (ESI) (Waters) in both positive and negative ionization mode with a pooled quality control (QC) sample injected for every 14th sample as previously described^47^.

The raw UPLC-MS data were converted to CDF files using the DataBridge software included in Masslynx (Waters) and were pre-processed using MZmine (version 2.35)^48^. Data tables were generated comprising m/z, retention time and intensity (peak height) for each feature in every sample. The pre-processed data were subsequently filtered in Matlab R2014b (The MathWorksInc., Natick, MA) by removing features present in blanks, duplicates, potential isotopes, features detected in less than 50 % of samples, and early and late eluting features (retention time < 0.3 min or > 6.45 min). The data were normalized to the total intensity. Finally, the data were filtered using the pooled QC samples; features with coefficient of variation above 0.2 in the QC samples were excluded. The CV% of the metabolites in the QC samples measured by UPLC-MS in positive (4612 features) and negative (529 features) ionisation mode was on average 10% and 11%, respectively.

The accurate masses of the discriminating features measured by UPLC-MS were searched for putative molecules in the METLIN^49^ and HMDB^50^ databases. The metabolites were identified according to the four different levels described by the Metabolomics Standard Initiative;^51^ metabolites confirmed by an authentic standard (Level I), metabolites confirmed based on a comparison of MS/MS fragmentation pattern compared with those found in databases and earlier literature (Level II), metabolites with similarities to published fragmentation patterns (Level III), and unknown compounds (Level IV).

Valine, leucine and isoleucine were confirmed by authentic standards obtained from Sigma Aldrich.

# References

1. Hecht DW. Anaerobes: Antibiotic resistance, clinical significance, and the role of susceptibility testing. Anaerobe [Internet] 2006; 12:115–21. Available from: http://www.sciencedirect.com/science/article/pii/S1075996405001319

2. Leclercq R, Cantón R, Brown DFJ, Giske CG, Heisig P, MacGowan AP, Mouton JW, Nordmann P, Rodloff AC, Rossolini GM, et al. EUCAST expert rules in antimicrobial susceptibility testing. Clin Microbiol Infect [Internet] 2013; 19:141–60. Available from: http://dx.doi.org/10.1111/j.1469-0691.2011.03703.x

3. Hollenbeck BL, Rice LB. Intrinsic and acquired resistance mechanisms in enterococcus. Virulence [Internet] 2012; 3:421–569. Available from: http://dx.doi.org/10.4161/viru.21282

4. Marchand-Austin A, Rawte P, Toye B, Jamieson FB, Farrell DJ, Patel SN. Antimicrobial susceptibility of clinical isolates of anaerobic bacteria in Ontario, 2010-2011. Anaerobe 2014; 28:120–5.

5. Lozniewski A, Labia R, Haristoy X, Mory F. Antimicrobial susceptibilities of clinical Desulfovibrio isolates. Antimicrob Agents Chemother 2001; 45:2933–5.

6. Brook I, Wexler HM, Goldstein EJ. Antianaerobic antimicrobials: spectrum and susceptibility testing. Clin Microbiol Rev [Internet] 2013; 26:526–46. Available from: http://www.ncbi.nlm.nih.gov/pubmed/23824372

7. Delgado S, Flórez AB, Mayo B. Antibiotic Susceptibility of Lactobacillus and Bifidobacterium Species from the Human Gastrointestinal Tract. Curr Microbiol [Internet] 2005; 50:202–7. Available from: http://dx.doi.org/10.1007/s00284-004-4431-3

8. Brook I. Spectrum and treatment of anaerobic infections. J Infect Chemother 2016; 22:1–13.

9. Nakao K, Tanaka K, Ichiishi S, Mikamo H, Shibata T, Watanabe K. Susceptibilities of 23 Desulfovibrio isolates from humans. Antimicrob Agents Chemother 2009; 53:5308–11.

10. Lofmark S, Edlund C, Nord CE. Metronidazole is still the drug of choice for treatment of anaerobic infections. Clin Infect Dis 2010; 1:647939.

11. Goldstein EJ, Tyrrell KL, Citron DM. Lactobacillus species: taxonomic complexity and controversial susceptibilities. Clin Infect Dis 2015; 15.

12. Moubareck C, Gavini F, Vaugien L, Butel MJ, Doucet-Populaire F. Antimicrobial susceptibility of bifidobacteria. J Antimicrob Chemother 2005; 55:38–44.

13. Zerbino DR. Using the Velvet de novo assembler for short-read sequencing technologies. Curr Protoc Bioinforma 2010; 11.

14. Harris HMB, Bourin MJB, Claesson MJ, O’Toole PW. Phylogenomics and comparative genomics of Lactobacillus salivarius, a mammalian gut commensal. Microb Genom 2017; 3.

15. Altschul SF, Gish W, Miller W, Myers EW, Lipman DJ. Basic local alignment search tool. J Mol Biol 1990; 215:403–10.

16. Maidak BL, Olsen GJ, Larsen N, Overbeek R, McCaughey MJ, Woese CR. The Ribosomal Database Project (RDP). Nucleic Acids Res 1996; 24:82–5.

17. Kaiser JC, Heinrichs DE. Branching out: Alterations in bacterial physiology and virulence due to branched-chain amino acid deprivation. MBio2018; 9.

18. Jia B, Raphenya AR, Alcock B, Waglechner N, Guo P, Tsang KK, Lago BA, Dave BM, Pereira S, Sharma AN, et al. CARD 2017: expansion and model-centric curation of the comprehensive antibiotic resistance database. Nucleic Acids Res 2017; 45:D566–73.

19. Chen L, Yang J, Yu J, Yao Z, Sun L, Shen Y, Jin Q. VFDB: a reference database for bacterial virulence factors. Nucleic Acids Res 2005; 33.

20. Tanoue T, Morita S, Plichta DR, Skelly AN, Suda W, Sugiura Y, Narushima S, Vlamakis H, Motoo I, Sugita K, et al. A defined commensal consortium elicits CD8 T cells and anti-cancer immunity. Nature [Internet] 2019 [cited 2020 Nov 7]; 565:600–5. Available from: https://pubmed.ncbi.nlm.nih.gov/30675064/

21. Ndongo S, Dubourg G, Bittar F, Sokhna C, Fournier PE, Raoult D. Marseillibacter massiliensis gen. nov., sp. nov., a new bacterial genus isolated from the human gut. New Microbes New Infect 2016; 16:30–1.

22. Mashima I, Kamaguchi A, Miyakawa H, Nakazawa F. Veillonella tobetsuensis sp. nov., an anaerobic, gram-negative coccus isolated from human tongue biofilms. Int J Syst Evol Microbiol [Internet] 2013; 63:1443–9. Available from: http://www.ncbi.nlm.nih.gov/pubmed/22843723

23. Ahn S, Jin TE, Chang DH, Rhee MS, Kim HJ, Lee SJ, Park DS, Kim BC. Agathobaculum butyriciproducens gen. nov. &nbsp;sp. nov., a strict anaerobic, butyrate-producing gut bacterium isolated from human faeces and reclassification of Eubacterium desmolans as Agathobaculum desmolans comb. nov. Int J Syst Evol Microbiol 2016; 66:3656–61.

24. Takada T, Watanabe K, Makino H, Kushiro A. Reclassification of Eubacterium desmolans as Butyricicoccus desmolans comb. nov., and description of Butyricicoccus faecihominis sp. nov., a butyrate-producing bacterium from human faeces. Int J Syst Evol Microbiol 2016; 66:4125–31.

25. Elsayed S, Zhang K. Human infection caused by Clostridium hathewayi. Emerg Infect Dis [Internet] 2004; 10:1950–2. Available from: http://www.ncbi.nlm.nih.gov/pubmed/15550205

26. Kaur S, Yawar M, Kumar PA, Suresh K. Hungatella effluvii gen. nov., sp. nov., an obligately anaerobic bacterium isolated from an effluent treatment plant, and reclassification of Clostridium hathewayi as Hungatella hathewayi gen. nov., comb. nov. Int J Syst Evol Microbiol 2014; 64:710–8.

27. Udaondo Z, Duque E, Ramos JL. The pangenome of the genus Clostridium. Env Microbiol [Internet] 2017; 19:2588–603. Available from: http://www.ncbi.nlm.nih.gov/pubmed/28321969

28. La Reau AJ, Meier-Kolthoff JP, Suen G. Sequence-based analysis of the genus Ruminococcus resolves its phylogeny and reveals strong host association. Microb Genom [Internet] 2016; 2:e000099. Available from: http://www.ncbi.nlm.nih.gov/pubmed/28348838

29. Lawson PA, Finegold SM. Reclassification of Ruminococcus obeum as Blautia obeum comb. nov. Int J Syst Evol Microbiol 2015; 65:789–93.

30. Liu C, Finegold SM, Song Y, Lawson PA. Reclassification of Clostridium coccoides, Ruminococcus hansenii, Ruminococcus hydrogenotrophicus, Ruminococcus luti, Ruminococcus productus and Ruminococcus schinkii as Blautia coccoides gen. nov., comb. nov., Blautia hansenii comb. nov., Blautia hydroge. Int J Syst Evol Microbiol 2008; 58:1896–902.

31. Carlier JP, Bedora-Faure M, K’Ouas G, Alauzet C, Mory F. Proposal to unify Clostridium orbiscindens Winter et al. 1991 and Eubacterium plautii (Seguin 1928) Hofstad and Aasjord 1982, with description of Flavonifractor plautii gen. nov., comb. nov., and reassignment of Bacteroides capillosus to Pseudoflavonifrac. Int J Syst Evol Microbiol [Internet] 2010; 60:585–90. Available from: http://www.ncbi.nlm.nih.gov/pubmed/19654357

32. Kim MS, Roh SW, Bae JW. Bacteroides faecis sp. nov., isolated from human faeces. Int J Syst Evol Microbiol [Internet] 2010; 60:2572–6. Available from: http://www.ncbi.nlm.nih.gov/pubmed/20008108

33. Zhong Z, Zhang W, Song Y, Liu W, Xu H, Xi X, Menghe B, Zhang H, Sun Z. Comparative genomic analysis of the genus Enterococcus. Microbiol Res [Internet] 2017; 196:95–105. Available from: http://www.ncbi.nlm.nih.gov/pubmed/28164795

34. Poyart C, Quesne G, Trieu-Cuot P. Taxonomic dissection of the Streptococcus bovis group by analysis of manganese-dependent superoxide dismutase gene (sodA) sequences: reclassification of “Streptococcus infantarius subsp. coli” as Streptococcus lutetiensis sp. nov. and of Streptococcus bov. Int J Syst Evol Microbiol 2002; 52:1247–55.

35. Chattaway MA, Schaefer U, Tewolde R, Dallman TJ, Jenkins C. Identification of Escherichia coli and Shigella Species from Whole-Genome Sequences. J Clin Microbiol 2017; 55:616–23.

36. Devanga Ragupathi NK, Muthuirulandi Sethuvel DP, Inbanathan FY, Veeraraghavan B. Accurate differentiation of Escherichia coli and Shigella serogroups: challenges and strategies. New Microbes New Infect [Internet] 2018; 21:58–62. Available from: http://www.ncbi.nlm.nih.gov/pubmed/29204286

37. Fukushima M, Kakinuma K, Kawaguchi R. Phylogenetic analysis of Salmonella, Shigella, and Escherichia coli strains on the basis of the gyrB gene sequence. J Clin Microbiol [Internet] 2002; 40:2779–85. Available from: http://www.ncbi.nlm.nih.gov/pubmed/12149329

38. Chung WS, Walker AW, Louis P, Parkhill J, Vermeiren J, Bosscher D, Duncan SH, Flint HJ. Modulation of the human gut microbiota by dietary fibres occurs at the species level. BMC Biol [Internet] 2016; 14:3. Available from: http://www.ncbi.nlm.nih.gov/pubmed/26754945

39. Ntemiri A, Chonchuir FN, O’Callaghan TF, Stanton C, Ross RP, O’Toole PW. Glycomacropeptide Sustains Microbiota Diversity and Promotes Specific Taxa in an Artificial Colon Model of Elderly Gut Microbiota. J Agric Food Chem 2017; 65:1836–46.

40. Klindworth A, Pruesse E, Schweer T, Peplies J, Quast C, Horn M, Glockner FO. Evaluation of general 16S ribosomal RNA gene PCR primers for classical and next-generation sequencing-based diversity studies. Nucleic Acids Res 2013; 41:28.

41. Magoc T, Salzberg SL. FLASH: fast length adjustment of short reads to improve genome assemblies. Bioinformatics 2011; 27:2957–63.

42. Caporaso JG, Kuczynski J, Stombaugh J, Bittinger K, Bushman FD, Costello EK, Fierer N, Pena AG, Goodrich JK, Gordon JI, et al. QIIME allows analysis of high-throughput community sequencing data. Nat Methods [Internet] 2010; 7:335–6. Available from: http://www.ncbi.nlm.nih.gov/pubmed/20383131

43. Martin M. Cutadapt Removes Adapter Sequences From High-Throughput Sequencing Reads. EMBnet.journal 2011; 17:10–2.

44. Edgar RC. Search and clustering orders of magnitude faster than BLAST. Bioinformatics [Internet] 2010; 26:2460–1. Available from: http://www.ncbi.nlm.nih.gov/pubmed/20709691

45. Schloss PD, Westcott SL, Ryabin T, Hall JR, Hartmann M, Hollister EB, Lesniewski RA, Oakley BB, Parks DH, Robinson CJ, et al. Introducing mothur: open-source, platform-independent, community-supported software for describing and comparing microbial communities. Appl Env Microbiol [Internet] 2009; 75:7537–41. Available from: http://www.ncbi.nlm.nih.gov/pubmed/19801464

46. Allard G, Ryan FJ, Jeffery IB, Claesson MJ. SPINGO: a rapid species-classifier for microbial amplicon sequences. BMC Bioinformatics [Internet] 2015; 16:324. Available from: http://www.ncbi.nlm.nih.gov/pubmed/26450747

47. Andersen MB, Kristensen M, Manach C, Pujos-Guillot E, Poulsen SK, Larsen TM, Astrup A, Dragsted L. Discovery and validation of urinary exposure markers for different plant foods by untargeted metabolomics. Anal Bioanal Chem 2014; 406:1829–44.

48. Pluskal T, Castillo S, Villar-Briones A, Oresic M. MZmine 2: modular framework for processing, visualizing, and analyzing mass spectrometry-based molecular profile data. BMC Bioinformatics 2010; 11:1471–2105.

49. Smith CA, O’Maille G, Want EJ, Qin C, Trauger SA, Brandon TR, Custodio DE, Abagyan R, Siuzdak G. METLIN: a metabolite mass spectral database. Ther Drug Monit 2005; 27:747–51.

50. Wishart DS, Jewison T, Guo AC, Wilson M, Knox C, Liu Y, Djoumbou Y, Mandal R, Aziat F, Dong E, et al. HMDB 3.0--The Human Metabolome Database in 2013. Nucleic Acids Res 2013; 41:17.

51. Sumner LW, Amberg A, Barrett D, Beale MH, Beger R, Daykin CA, Fan TW, Fiehn O, Goodacre R, Griffin JL, et al. Proposed minimum reporting standards for chemical analysis Chemical Analysis Working Group (CAWG) Metabolomics Standards Initiative (MSI). Metabolomics 2007; 3:211–21.
