# Supplementary material for: A synthetic consortium of 100 gut commensals modulates the composition and function in a colon model of the microbiome of elderly subjects
Source: Gut Microbes. 2021 May 16;13(1):1919464. doi: 10.1080/19490976.2021.1919464 (PMC8128205; doi:10.1080/19490976.2021.1919464)
Supplement: Supplemental Material [file KGMI_A_1919464_SM3620.zip › Supplementary information/Supplementary material_REVISED.docx]

# Supplementary Material

**Supplementary Table 1: Selection of gut microbial species.** **a**. List of genera and species in the MCC and references showing their presence in the core and among the most abundant taxa in the human microbiota. Studies that describe close relative taxa to those present in the collection are indicated with the reference number in brackets. References showed below. The classification of representative isolates of each species within the iBBiG-defined OTU groups (ref. 7) are indicated: Co, Core; RC, Reduced Core; Co-RC, Core-Reduced Core; DA, Diversity Associated; Co-DA, Core-Diversity Associated; Co-DA-LA, Core-Diversity Associated-Long-stay Associated; LA-RC, Long-stay Associated-Reduced Core; Ua, Unassigned. Last column shows species selected for the MCC100 consortium. **b**. Revised studies describing the most prevalent and abundant microbial taxa in the human gut microbiota. The number of donors analysed, the criterion to define the taxa present in the core microbiota, the number of defined taxa and the number of taxa present in the MCC are indicated for each study. References provided below. The taxonomic assignation corrected by metaref.org (consulted on 2015) was used for the unknown species in ref. 11.

**Supplementary Table S2:** Summary of RAPD-PCR results showing the number of MCC isolates analysed per bacterial group, the number of RAPD-PCR patterns obtained with the primer used and the number of different strains segregated per group. Isolates were grouped for the pattern comparisons as indicated in the first column.

**Supplementary Table S3. List of microbial isolates comprising the synthetic consortium Microbiome Culture Collection 100 (MCC100).** Initial assignation to closest species and sequence identity (%) obtained by BLASTn search of the 16S rRNA amplicon against the NCBI database (consulted on 2015), and phylum, family and genus assigned by RDP Browse Hierarchy tool are indicated (columns B-F). Table shows media used for isolation and routine culture (columns G-H); species ID after taxonomic analysis (column I); and volume and OD600 values of the individual cultures used to prepare 1000 mL of MCC100 inoculum (columns J-K).

**Supplementary Table S4**: Assembly statistics and quality assessments of the MCC100 strains genomes. Quality was assessed by BLAST search (tblastn) the 40 universal marker genes (Wu et al 2013) against the contigs of each genome. Classification in the “Most wanted” taxa priority list of the Human Microbiome Project (HMP) is listed in column M.

**Supplementary Table S5: Antimicrobial susceptibility of the MCC100 bacterial strains. a.** MIC values (mg L-1) of 7 antibiotics tested against the 99 MCC100 bacterial strains. MICs were determined by gradient strip method on Brucella Agar plates supplemented with 5% defibrinated blood, 5 mg L-1 hemin and 1 mg L-1 vitamin K (except for strain MCC585 that was tested on YCFA) under anaerobic conditions. The susceptibility profile of the strains was interpreted according to EUCAST breakpoints and is showed by colour shading: susceptibility (green), intermediate resistance (yellow), resistance (red) and intrinsic resistance (blue). While resistance refers to the ability to resist the antimicrobial activity which might have been acquired through mutation or genetic material exchange, intrinsic resistance indicates that resistance trait is naturally inherent in all the strains of a bacterial group. The intrinsic resistances are documented in the references indicated below the table. Vancomycin was not tested in Gram-negative bacteria since they are naturally resistant and the test is not required by EUCAST. **b.** Number of strains that can be inhibited by one or two combined antibiotics used in Table S5a, assuming natural vancomycin resistance in the non-tested strains. Conditions that inhibit all the bacteria are shadowed in grey. **c.** Inhibition diameter values (mm) of additional antibiotics tested in the facultative aerobic strains by EUCAST disk diffusion method on Mueller-Hinton agar plates in aerobic conditions (enterococci and enterobacteria) or Muller-Hinton agar supplemented with 5% defibrinated horse blood and 20 mg L-1 β-NAD in microaerophilic conditions (streptococci). Antibiotic dick content (µg) is provided. Quinupristin-dalfopristin was not tested in *Enterococcus faecalis* since it is naturally resistant and the test is not required by EUCAST. Results were interpreted following EUCAST guidelines and susceptibility levels are indicated by colour shading as in Table S5a.

**Supplementary Table S6:** **Putative antibiotic resistance genes, virulence factors, and bacteriocins annotated in the genomes of the MCC100 strains.** Predicted amino acid sequences were aligned with BLASTP against the Comprehensive Antibiotic Resistance Database (CARD), Virulence Factor Database (VFDB) and the bacteriocin database (BAGEL3), respectively. **a.** Number of copies of putative antibiotic resistance genes found across MCC100 genomes. Genes are grouped by the drug class they confer resistance to. Antimicrobials whose MIC was determined are indicated in square brakets in each drug class. Correlation with resistance phenotype was found for some clindamycin and benzylpenicillin resistant strains and is indicated in bold and with a border. **b.** Number of copies of putative virulence factors found across MCC100 genomes. **c.** Putative bacteriocins found across MCC100 genomes.

**Supplementary Table S7. List of species unique to each group when comparing the samples supplemented with the MCC100 with their corresponding control groups.** (A taxon is considered as present if detected in at least 50% of the samples within a group)

**Supplementary Table S8. Metabolites identified and annotated by UPLC-MS. a**. Negative mode. **b.** Positive mode.

**Supplementary Table S9: Characteristics of the volunteers recruited for faecal sample donation.**

**Supplementary Table S10: Culture media used in this study.** Media 1 to 31 were used for the isolation and routinely growth of gut microorganisms. Agar (20 g) was added when necessary. Supplementation with antibiotics was based on Rettedal et al (2014) unless other reference stated. Medium 32 was used for fermentation experiments. References are indicated below the table.

**Supplementary Table S11.** SRA accession number of 16S rRNA gene sequence data.

**Supplementary Table S12. OTUs classification.** Classification of representative sequences for each OTU was carried out using mothur against the RDP database (release 11) and the species classifier SPINGO version 1.2 with default parameters. avg. abun. =average abundance; min abun.=minimum abundance; max abun.=maximum abundance; nb.=number of samples in which the OTU is detected.

**Supplementary Figure S1:** Example of RAPD-PCR profiles obtained with the primers OPL5, M13 and 1254. Each lane corresponds to an isolate of the indicated closest species. Lane M: 1-kb DNA molecular mass ladder (Bioline).

**Supplementary Figure S2:** Example of an UPGMA dendrogram derived from a comparison of the RAPD-PCR patterns obtained with primer M13 for the isolates of the genus *Bacteroides*. Dendrograms were performed using Number of different bands as similarity coefficient. Asterisks indicate the strains selected for the artificial consortium MCC100.

**Supplementary Figure S3:** Phylogenetic positioning of the MCC100 strains among 74 reference strains. Phylogenetic maximum likelihood tree using the Generalized Time-Reversible model with CAT approximation with 20 rate categories inferred from the 16S rRNA gene. The tree is rooted on the domain Archaea for illustrative purposes. Local support values superior or equal to 70% are displayed.

**Supplementary Figure S4**: Principal coordinate analysis (PCoA) differentiates microbiota patterns of elderly donors living in the community (red) and long-stay care (blue) on weighted (left) and unweighted (right) UniFrac distance matrices.

**Supplementary Figure S5:** (a) Alpha-diversity indexes of the gut microbiota of elderly donors living in the community and long-stay care. (b) Presence of MCC100 species across donors microbiota. BLAST results of the MCC100 16S rRNA gene full-length sequences against the V3/V4 16S rRNA gene sequences of the faecal samples were filtered at 98.7% identity and 90% coverage. MCC100 taxa with one hit or greater were considered as present in the sample. Statistically significant differences were determined using Mann-Whitney (one-tailed) (**∙** exact p-value=0.05).

**Supplementary Figure S6:** Alpha-diversity indexes of (a) synthetic consortium MCC100 inoculum and fermentation samples of the MCC100 alone (time 0 and time 3); and (b)aggregated faecal fermentations supplemented with MCC100 (green) or control (red) at time 0 and after 3 days of culture (time 3). Statistically significant differences between control and MCC100 supplemented fermentation groups were determined using Mann-Whitney (one-tailed) (* p-value < 0.05).

**Supplementary Figure S7:** Bacterial community profiles at family level for control and MCC100 supplemented microbiota typesand MCC100 fermentation alone at time 0 and time 3. Only dominant bacterial species (relative abundance ≥ 1%) are displayed. Species within the same phylum are indicated by different shades of the same colour.

**Supplementary Figure S8:** (a) Fold differences in relative abundance of bacterial species showing significant differences between time 0 and time 3 for control and MCC100 supplemented fermentations. Bacterial species were classified as dominant (relative abundance ≥ 1%), low abundant (relative abundance between 0.1 and 1%) or rare (relative abundance ≤ 0.1%). Statistically significant differences were determined using Kruskal-Wallis (p-adjust<0.01) + Dunn’s test (* *p*<0.05 ** *p*<0.005). (b) Shared and unique bacterial species detected in 16S rRNA gene sequencing analysis at time 0 and time 3 in MCC100 supplemented (green) and control (red) fermentations (species that were present in both technical replicates and in at least 50% of the samples in each group). (c) MCC100 taxa identified in MCC100 supplemented and control fermentations at time 0 and time 3 by BLAST searches of the MCC100 16S rRNA gene full-length sequences against the V3/V4 16S rRNA gene sequences.

**Supplementary Figure S9:** Association between the relative abundance of annotated metabolites and the relative abundance of bacterial species. Spearman’s rank correlations were calculated between the relative abundance of metabolites putatively identified by UPLC-MS (negative (-) and positive (+) ionization modes) and relative abundance of bacterial species in control and MCC100 supplemented fermentations at time 3. Unsupervised hierarchical clustering was applied on metabolites and bacterial species. The BCAA leucine, valine and isoleucine are indicated in a square.
